# Exploration of the anticancer efficacy of a novel 1,3-thiazole analog in an ehrlich ascites carcinoma model: *in vivo* and *in silico* insights into hepatorenal protective potentials *via* the modulation of apoptosis, oxidative stress and inflammation†

**DOI:** 10.1039/d5ra01014d

**Published:** 2025-06-13

**Authors:** Mohammed El Behery, Dina M. Abo-Elmatty, Maha Alsunbul, Yassmina I. Mohey El-Deen, Doaa I. Mohamed, Emad Rashad Sindi, Maha H. Hashem, Ebtesam Al-Olayan, Ibrahim Abdel Aziz Ibrahim, Ghazi A. Bamagous, Suraj Mali, Essa M. Saied

**Affiliations:** a The Division of Biochemistry, Chemistry Department, Faculty of Science, Port Said University Port Said 42526 Egypt elbehery@sci.psu.edu.eg; b Biochemistry Department, Faculty of Pharmacy, Suez Canal University Ismailia 415222 Egypt dina_aboelmatty@pharm.suez.edu.eg; c Department of Pharmaceutical Sciences., College of Pharmacy, Princess Nourah Bint Abdulrahman University P.O. Box 84428 Riyadh 11671 Saudi Arabia Maalsonbel@pnu.edu.sa; d Pharmaceutical Organic Chemistry Department, Faculty of Pharmacy, Suez Canal University Ismailia 41522 Egypt yassmina.ibrahim@icloud.com; e Department of Clinical Pharmacology, Faculty of Medicine, Ain Shams University Cairo 11566 Egypt doaapharma@med.asu.edu.eg maha_hussein@med.asu.edu.eg; f Department of Basic Medical Sciences, College of Medicine, University of Jeddah Jeddah 23890 Saudi Arabia ersindi@uj.edu.sa; g Department of Zoology, College of Science, King Saud University Riyadh Saudi Arabia eolayan@ksu.edu.sa; h Department of Pharmacology and Toxicology, Faculty of Medicine, Umm Al-Qura University Makkah Saudi Arabia iamustafa@uqu.edu.sa gabamagous@uqu.edu.sa; i School of Pharmacy, D.Y. Patil University (Deemed to be University) Sector 7, Nerul 400706 Navi Mumbai India suraj.mali@dypatil.edu; j Chemistry Department, Faculty of Science, Suez Canal University Ismailia 41522 Egypt essa.saied@science.suez.edu.eg; k Institute for Chemistry, Humboldt Universität zu Berlin 12489 Berlin Germany saiedess@hu-berlin.de

## Abstract

Thiazoles, as a class of compounds, offer a diverse array of analogs that are pivotal in the rational design of anticancer agents. Recently, we reported a novel 1,3-thiazole analog, 2-(1-(2-(4-(4-bromophenyl)thiazol-2-yl)hydrazinylidene)ethyl)phenol (BTHP), that exhibited potential cytotoxic activity toward breast cancer cells. In the present study, we extended our investigations to explore the anticancer potential of BTHP in Ehrlich Ascites Carcinoma (EAC)-administrated female Swiss albino mice. Our findings revealed that, compared with the control group, the expression levels of antioxidant enzymes significantly decreased in the EAC-induced model group, while the level of lipid peroxidation substantially increased. Furthermore, the administration of EAC impaired hepatorenal function, as indicated by a significant increase in serum aspartate aminotransferase (AST), alanine aminotransferase (ALT), creatinine, and urea levels and a decrease in total protein and albumin levels. EAC-induced renal and hepatic damage was accompanied by elevated expression of proinflammatory biomarkers (TGF-β, NFκB, and IL6 genes) and altered serum apoptotic signaling, including reduced p53, Bax, caspase-3, and cytochrome c levels, alongside increased Bcl-2 expression. Interestingly, administration of BTHP (5 mg per kg per day, 14 days) significantly mitigated the viable EAC cell count (38%) and enhanced lifespan (131.25%) compared to untreated EAC-bearing mice. Furthermore, compared with the EAC-induced model group, the BTHP-treated EAC-induced group exhibited significantly attenuated lipid peroxidation levels and enhanced antioxidant enzyme activity (superoxide dismutase, glutathione, and catalase). Moreover, BTHP improved hepatorenal function by restoring serum ALT, AST, urea, creatinine, albumin, and total protein levels. Remarkably, BTHP reversed the apoptotic dysregulation observed in the EAC model, significantly elevating p53, Bax, caspase-3, and cytochrome c levels while suppressing Bcl-2 expression. Anti-inflammatory effects were further evidenced by diminished NFκB, TGF-β, and IL6 expression in liver and kidney tissues. Histological examinations confirmed BTHP's efficacy in attenuating EAC-induced renal and hepatic damage, preserving structural integrity. Finally, detailed molecular modeling investigations revealed that BTHP exhibits a pronounced binding affinity toward key protein targets associated with the observed anticancer activity. Overall, this study underscores the anticancer efficacy of BTHP through the regulation of antioxidant, proinflammatory, and apoptotic biomarkers, highlighting its protective effects on hepatorenal function and its therapeutic potential.

## Abstract 2

Delving into the multifaceted therapeutical potential of BTHP as a novel 1,3-thiazole-based anticancer agent.

## Graphical abstract

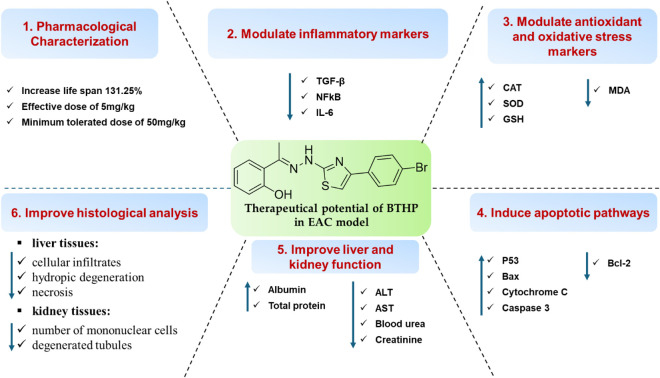

## 1. Introduction

Cancer remains one of the most common significant causes of morbidity and mortality worldwide, causing approximately 1 in every 6 deaths globally and nearly 10 million deaths in 2022.^1,2^ The cancer mortality rate is predicted to double to nearly 20 million deaths by 2050.^3,4^ The estimated number of incident cases of cancer worldwide in 2050 is more than 35M cases compared with 20M new cases in 2022. Lung, breast, bronchus, prostate, and colorectal cancers are the most common cancers worldwide, accounting for 50% of all newly diagnosed cases and cancer-related deaths.^5,6^ The routine dealing with cancer includes local treatments such as surgery or in combination with systemic cancer therapies such as chemotherapy, hormone therapy, and immunotherapy.^7–9^ However, the use of both conventional local and systemic therapies is associated with critical hurdles, such as specificity and the emergence of cancer drug resistance due to genetic mutations, enhanced drug efflux, and other molecular mechanisms.^10,11^ Therefore, there is urgent need for the discovery of novel potent natural or synthetic anticancer molecules with new mechanisms and fewer side effects.^12–14^

Heterocycles are among the most bioactive molecules since they have a vast range of considerable activity, including antimicrobial, antiviral, antioxidant, and anticancer effects.^15–17^ Heterocyclic-based components are commonly present in a wide range of bioactive molecules, most of which are of natural origin, including alkaloids, vinblastine, and reserpine, in addition to several other medications, such as cephalosporin and penicillin.^18,19^ The primary class of chemical compounds among biologically active complexes, natural products, and chemicals frequently used in medicinal chemistry is thought to be heterocyclic compounds containing nitrogen and sulfur atoms. Indeed, both sulfur and nitrogen play major roles in biological systems, which proves the potential biological significance of nitrogen sulfur-containing heterocycles.^16,20,21^ The family of sulfur–nitrogen heterocycles, including thiazine, piperazine–benzothiazines, morpholine-benzothiazines, benzothiazines, and thiazole, is a significant platform for rational drug design of anticancer agents with potent therapeutic effects.^16,20,22–24^

Thiazole, or 1,3-thiazole, is a 5-membered heterocyclic compound that contains both sulfur and nitrogen. The thiazole ring is a core component of common pharmacologically active molecules such as vitamin B, penicillin-like antibiotics, pramipexole, meloxicam, and nizatidine.^23,25–27^ Several thiazole-based heterocyclic compounds, such as vosaroxin, dasatinib, epothilones, bleomycin, ixabepilone, and tiazofurin, have been authorized as anticancer agents.^28,29^ Different cancer cell death pathways have been reported for the action of thiazole scaffolds, such as kinase, polymerase, microtubular inhibitors, topoisomerase II, and vascular endothelial growth factor receptor-2 (VEGFR-2).^24,25,27,30,31^ The anticancer activities of thiazole compounds may also be attributed to their potent antioxidant effects which are associated with their structural diversity.^32–34^ A series of thiazole compounds displayed remarkable metal chelating and free radical scavenging activities that prevent damage to membrane and cellular proteins and lipids.^35^ These activities were attributed to the ability of thiazole compounds to inhibit nitric oxide radical scavenging, ferric ion reduction, hydrogen peroxide, and lipid peroxidation compared with antioxidant standards such as ascorbic acid.^36,37^ By neutralizing free radicals and reducing oxidative stress, antioxidants play a role in DNA damage repair, inhibiting the growth and cell division rates of abnormal cells and decreasing mutagenicity.^38^

The Ehrlich ascites carcinoma (EAC) model, which is a widely recognized mouse model of mammary adenocarcinoma, provides significant and useful knowledge of mammary tumors in mice.^39^ It offers a valuable platform for examining the advancement of tumors, metastasis, and the effectiveness of treatments in controlled laboratory conditions.^40^ This model also provides a valuable understanding of the intricate interaction between cancer cells and their microenvironment, a critical factor in the development of potential treatment approaches for cancers.^41,42^

**Fig. 1:**
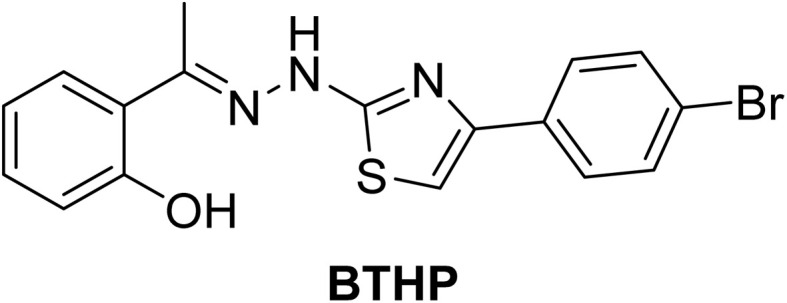
Chemical structure of 2-(1-(2-(4-(4-bromophenyl)thiazol-2-yl)hydrazinylidene)ethyl)phenol (BTHP).

Recently, our group reported the synthesis and cytotoxic activity of a set of novel 1,3-thiazole analogs.^24^ Among the examined compounds, 2-(1-(2-(4-(4-bromophenyl)thiazol-2-yl)hydrazinylidene)ethyl)phenol (BTHP) exhibited potential antiproliferative activity toward MDA-MB-231 and MCF-7 breast cancer cells, which was associated with its ability to target VEGFR-2 (Fig. 1). Encouraged by these findings and our interest in discovering novel anticancer agents,^43–51^ in this study, we aimed to explore the therapeutic potential of BTHP as an anticancer agent *in vivo* in female EAC Swiss albino mice. Therefore, we explored the therapeutic potential of BTHP by assessing its lethal dose and effective dose as well as its ability to modulate oxidative stress, antioxidant enzymes, proinflammatory biomarkers, and apoptotic pathways. Furthermore, we assessed its hepatorenal protective activity by biochemically and histologically evaluating liver and kidney function and architecture. Finally, we performed detailed *in silico* studies of different targeted proteins to explore and confirm the mode of action associated with the observed anticancer activity.

## 2. Materials and methods

### 2.1 Chemicals and reagents

All chemicals and reagents used in this study were purchased from Sigma-Aldrich Chemical Company (St. Louis, MO, USA). BTHP was synthesized and characterized as previously reported by our group, and the purity was confirmed by NMR, MS, and FT-IR analysis.^24^

### 2.2 Experimental animals

Adult female Swiss albino mice weighing 24–27 grams (total animal = 90) were collected from the Abo Rawash Culture Facility, Giza, Egypt. Ehrlich cells, derived from a spontaneous mammary adenocarcinoma that arose in female mice, require the estrogen and progesterone-rich hormonal environment of females for tumor establishment and growth. Male mice are unable to support the growth of Ehrlich ascites carcinoma due to the absence of the requisite hormonal milieu. Thus, the use of female mice ensures the relevance of this model to human hormone-positive breast cancer. Following the acquisition, the mice were reared in stainless steel mesh cages under constant room temperature and humidity with strict hygienic conditions at the Animal House (Suez Canal University, Faculty of Pharmacy). The standard mouse diet and water were provided in adequate amounts. These animals were allowed to adapt for one week (acclimatization period). This phase was crucial for facilitating the physiological adjustment of the mice, ensuring their readiness for subsequent *in vivo* experiments. The protocol used in this study was approved ethically by the Animal Ethical Committee of the Faculty of Pharmacy, Suez Canal University, Egypt (Approval No. 202111MA1).

### 2.3 EAC cells and tumor induction in mice

The initial inoculum of the EAC cells was acquired from the National Cancer Institute (Cairo, Egypt). The propagation and maintenance of these cells as ascites-derived tumors were carried out by the intraperitoneal (IP) injection of 2.5 × 10^6^ viable cells per animal in the peritoneal cavity of the mice.^52,53^ For *in vitro* culture, EAC cells were grown in RPMI-1640 medium (Thermo Fisher Scientific, CAT. NO. 11875093, Waltham, MA, USA), which was supplemented with glucose, 2 mM l-glutamine, 10% fetal calf serum (FCS), and 1% penicillin–streptomycin (v/v). These cells were maintained in a humidified incubator at 37 °C with 5% CO_2_, and the medium was refreshed every 2–3 days. When the cells reached approximately 90% confluency, they were gently mixed, centrifuged at 200–300×*g* for 5–10 minutes to pellet the cells, and then resuspended in a fresh medium.^54^ The volume of EAC cells in the experimental group was determined with a graduated syringe, and then diluted with heparinized saline to determine the number of live cells using the trypan blue method.^55^ Briefly, 0.2 mL of EAC cells were mixed with 0.2 ml of 1% trypan blue solution (1 g of trypan blue in 100 ml of dH_2_O), and the resulting mixture was kept for 10 min at 37 °C.^56^ Subsequently, the cells were examined using a hemocytometer (Thoma type, Germany) under a light microscope at magnification ×40. The sum count of EAC cells was assessed utilizing the following equation:No. of cells per mL = no. of viable cells per 5 square × dilution factor × 10^4^

### 2.4 Determination of the lethal dose (LD)

The LD of BTHP was evaluated following the method used earlier by Meier and Theakston.^57^ Various doses of BTHP (in sterile saline solution, 0.9% NaCl) in the form of potassium salt (1–100 mg kg^−1^; 1–10, 30, 50, 100 mg kg^−1^) were injected intraperitoneally into Swiss albino mice in a final volume of 150 μL, and the controls were injected with saline alone (*n* = 3/group, total animal = 39). The mice were carefully observed for 48 hours to evaluate the lethal threshold. In the current study, the first group of mice was treated with BTHP at 1–10 mg per kg doses and the mice monitored for 48 h for any death or side effects. As no death was observed, a second group of mice was injected with 30, 50, and 100 mg per kg doses and subsequently observed for 48 h. This approach helps minimize the number of animals required for the study while ensuring reliable data collection across a wide range of doses.

### 2.5 Assessment of the effective dose

The effective *in vivo* dose of BTHP to reduce the volume and number of EAC cells was assessed following the methodology outlined by Crump *et al.*^58^ Five groups of mice (*n* = 3/group, total animal = 15) were initially administered EAC cells on the first day at a dosage of 2.5 × 10^6^ cells per mouse through intraperitoneal (IP) injection daily for 14 days. In the first group, G1, the EAC-induced group (ECA-control group), was treated with sterile saline solution (0.9% NaCl). The other four groups were subjected to varying doses of BTHP (in sterile saline solution, 0.9% NaCl) for 14 days: G2, ECA + BTHP (2.5 mg per kg per day); G3, ECA + BTHP (5 mg per kg per day); G4, ECA + BTHP (10 mg per kg per day); and G5, ECA + BTHP (15 mg per kg per day). During this time, the health and mortality of the mice were monitored daily until the conclusion of the experiment on the 14th day. For the assessment of EAC cell viability and counting, the trypan blue exclusion method was utilized as previously reported by Mclimans *et al.*^55^

### 2.6 Experimental animal design

Based on the assessment of the effective dose, we have selected a 5 mg per kg dose to assess the anticancer activity of BTHP in an animal model. Indeed, we selected 5 mg kg^−1^ as the minimal effective dose (MED) based on the pharmacokinetic, which exhibits the dose that displays a significant pharmacological effect (38% change) with minimal risk of toxicity and overdose to avoid adverse effects. Further, we considered the future possibility of exploring the BTHP in combination with anticancer therapy, which would be advantageous to use minimal doses of examined drugs. A total of 24 female Swiss albino mice, averaging 25–27 g in weight, were divided into four groups (*n* = 6/group, total animal = 24). The mice were maintained in an environment with controlled conditions, including a consistent 12-hour light–dark cycle and sufficient ventilation, providing a steady supply of fresh air. The ambient temperature was regulated within an optimal range to promote the animals' comfort and overall health. Cage hygiene was maintained through regular cleaning, and the mice were provided uninterrupted access to fresh water and standard laboratory chow, ensuring their well-being throughout the study period. The animals were grouped as follows:

• Group 1 (control group): mice in this group were administered intraperitoneal (IP) injections of sterile saline solution (0.9% NaCl) daily for 14 days.

• Group 2 (BTHP-treated group): mice in this group received daily I.P. injections of the potassium salt of BTHP (in sterile saline solution, 0.9% NaCl) at a dose of 5 mg per kg per day for 14 days.

• Group 3 (EAC-treated group): mice in this group were injected once with EAC cells (2.5 × 10^6^ cells per mouse) on the first day of the experiment, and the experiment ended on the 14th day.

• Group 4 (EAC + BTHP-treated group): mice were initially I.P. injected with EAC cells (2.5 × 10^6^ cells per mouse) on the first day of the experiment, followed by daily (I.P.) injections of the potassium salt of BTHP (in sterile saline solution, 0.9% NaCl) at 5 mg per kg per day for 14 days.

On the 14th day (end of the experiment), the viability and number of EAC cells in each group were reassessed using the trypan blue exclusion method.^55^ Additionally, the animals were fasted overnight and anesthetized *via* inhalation of light ether. The retro-orbital venous plexus was utilized to collect blood samples, and subsequently, the serum was separated from the blood by centrifuge at 3000 rpm. The obtained serum was stored at −80 °C for further serum biochemical investigations.^59^ After the mice were sacrificed, the liver and kidney tissues from each mouse were divided into two parts. For tissue homogenate preparation, the first part was maintained in phosphate-buffered saline (PBS, pH 7.4).^60^ The histopathological examinations were conducted utilizing the second part after fixation in a 10% formalin solution.

### 2.7 Assessment of animal lifespan

The impact of BTHP on the lifespan of mice bearing EAC was assessed by measuring the mean survival time (MST) and the percentage increase in lifespan (%ILS). After the treatment was completed, the effect of BTHP on tumor progression was assessed by monitoring the mortality of the animals (*n* = 6/group, total animal = 12). The mean survival time was determined, and the percentage increase in lifespan was calculated according to the methodology previously outlined following the formula:^61^
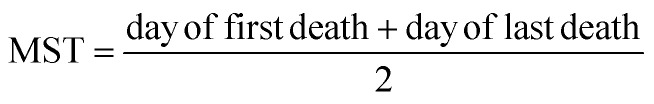

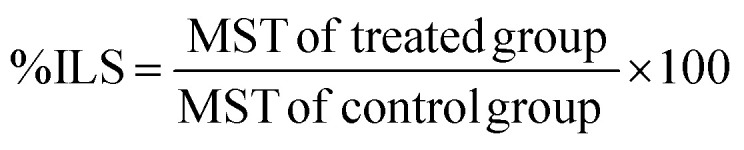


The *T*/*C* ratio was assessed following the formula:^62,63^
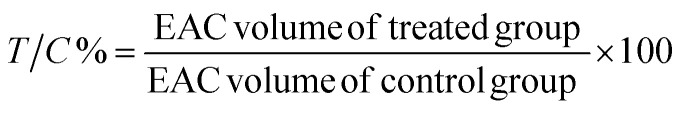


### 2.8 Assessment of antioxidant markers

#### 2.8.1 Determination of glutathione (GSH) levels

The concentration of reduced glutathione (GSH) was measured in the tissue homogenate following the protocol established by Beutler and Kelly.^64^ This method involves the reaction of GSH in a tissue homogenate with 5,5′-dithiobis (2-nitrobenzoic acid) (DTNB) to form a yellow-colored product (Abcam, USA, Cat. No. ab239727). The intensity of the color, which correlates directly with the GSH concentration, was quantified colorimetrically at 405 nm. This assay is sensitive to the redox state of the cell, reflecting the cellular capacity to counteract oxidative stress.

#### 2.8.2 Determination of superoxide dismutase (SOD) levels

Superoxide dismutase (SOD) activity, which is indicative of the body's defense against superoxide radicals, was measured in the tissue homogenate using a technique described by ref. 65. This assay relies on the SOD-mediated inhibition of the phenazine methosulfate-driven reduction of nitroblue tetrazolium dye to formazan, which can be measured spectrophotometrically (My BioSource, San Diego, CA, USA, Cat. No. MBS266897). The degree of inhibition of this reaction is proportional to the SOD activity in the tissue homogenate.

#### 2.8.3 Determination of catalase (CAT) levels

The activity of catalase, a key marker for hydrogen peroxide detoxification in cells, in the tissue homogenate was assessed colorimetrically as previously described.^66^ The assay involves the decomposition of hydrogen peroxide (H_2_O_2_) by catalase, followed by the detection of the remaining H_2_O_2_ in a reaction with peroxidase (HRP), 4-aminophenazone (AAP), and 3,5-dichloro-2-hydroxybenzenesulfonic acid (DHBS) (Abcam, USA, Cat. No. ab118184). The catalase concentration in the tissue homogenate intensity of is directly proportional to the resultant color. This assay provides crucial information about the ability of tissues to modulate oxidative stress through the rapid breakdown of hydrogen peroxide, a potentially harmful oxidizing agent.

#### 2.8.4 Determination of malondialdehyde (MDA) levels

To determine the concentration of MDA in the tissue homogenate, we employed the assay according to the manufacturer's instructions provided by the OxiSelect™ Thiobarbituric Acid Reactive Substances (TBARS) kit (Cell BIOLABS, USA; CAT No. STA-330-5).^67^ This assay relies on the formation of a red complex between MDA and thiobarbituric acid (TBA), which can be measured spectrophotometrically at 532 nm. Sample preparation involved combining the sample or standard solution with SDS lysis solution, TBA reagent, and BHT solution, followed by incubation at 95 °C. After cooling and centrifugation, the absorbance of the supernatant was measured, and the MDA concentration was calculated using a standard curve.

### 2.9 Assessment of the liver profile

A comprehensive evaluation of liver function was performed to quantify several key biochemical markers in the different experimental groups. Spectrophotometric determinations of the serum alanine aminotransferase (ALT) (Biomatik, Canada, Cat. No.: EKF58592) and aspartate aminotransferase (AST) (Abcam, USA, Cat. No.: ab263882) levels were performed. For AST, the oxaloacetate produced by glutamate oxaloacetate transaminase from aspartate was coupled with acetyl-CoA through citrate synthase to form citrate and CoA-SH. The resulting CoA-SH then reacted with DTNB (5,5′-dithio-bis(2-nitrobenzoic acid)), producing a chromophore with high absorbance at 412 nm, which was quantified spectrophotometrically.^45^ For ALT, the pyruvate produced from alanine by alanine aminotransferase is reduced by lactate dehydrogenase (LDH) to lactate with the concurrent oxidation of nicotinamide adenine dinucleotide phosphate (NAD)–hydrogen (NADH) to NAD^+^. The decrease in the NADH absorbance at 340 nm was detected.^45^ Additionally, we assessed the serum albumin (ALB) (Biomatik, Canada, Cat. No.: AB 10 10) and total protein (total protein; LS Bio, USA, Cat. No. LS-K272-100) levels to gain insight into the function of the liver. In this regard, the biuret method was employed to estimate the total serum protein concentration in the different experimental groups. Proteins that contain two or more peptide bonds react with copper ions to form a colored complex with an absorbance of *λ*_max_ = 454 nm, which is directly proportional to the protein concentration.^45,68^ Serum ALB levels were determined using the bromocresol green (BCG) method. This method relies on the high affinity of bromo-cresol green dye for albumin. The dye binds specifically to albumin and forms a complex that can be detected spectrophotometrically at *λ*_max_ = 628 nm.

### 2.10 Assessment of kidney profile

Similarly, to evaluate the impact of BTHP on renal function in ECA-induced female albino mice, a biochemical analysis was conducted. The blood urea concentration, a key indicator of renal function, in the serum was measured calorimetrically using an ELISA kit (MyBioSource, USA, Cat. No.: MBS2611085). The samples were combined with specific color reagents from the kit and incubated at room temperature for 30 minutes to allow a chromogenic reaction to occur. The urea concentration in the samples, which is directly proportional to the intensity of the resulting color, was then quantified by using a microplate reader at 450 nm. Additionally, the serum creatinine level, which is critical for assessing kidney filtration efficiency, was determined using a creatinine enzymatic assay kit (Elabscience, China, Cat. No.: E-BC-K188-M). The enzymatic assay involves sequential reactions of creatinine to creatine by creatininase and then creatine to sarcosine by creatine amidinohydrolase. Sarcosine is oxidized by sarcosine oxidase, yielding hydrogen peroxide. In the presence of peroxidase, hydrogen peroxide reacts with a chromogenic substrate to form a yellow dye. The intensity of this dye, which directly correlates with the creatinine concentration, was measured by its absorbance at 550 nm.

### 2.11 Assessment of the expression of proinflammatory markers in the liver and kidney

The levels of the transforming growth factor beta (TGF-β), interleukin-6 (IL-6), and nuclear factor-κB (NFKB) genes expressed in the liver and kidney tissues of the experimental models were quantified using real-time quantitative reverse transcription PCR (qRT-PCR) following the previously described protocol.^69^ RNA isolation was performed following the manufacturer's protocol, utilizing TRIzol™ (Thermo Fisher Scientific, Cat. No: 12183555) to lyse biological material, denature proteins, and maintain RNA integrity. Subsequently, RNA was purified using a silica-cartridge method. The isolated RNA was reverse transcribed into cDNA in a 20 μL reaction volume using RNase-free H_2_O. The reaction mixture consisted of sterile water (20 μL), total RNA (3 μL), reaction buffer (2.5 μL, 10×), reverse transcriptase (0.3 μL), oligo-dT primer (5 μL) and dNTPs (2.5 μL, 10 mM). The reaction mixture was allowed to incubate at 37 °C for 2 h in a thermal cycler and an additional 30 min at 65 °C before being stored at −20 °C. The qRT-PCR was conducted utilizing the iTaq universal SYBR® Green PCR Master Mix Kit (Bio-Rad, USA, Cat. No. 172-5150). The utilized primer pairs included (Table S1†): inducible MUS IL-6, forward GAGGATACCACTCCCAACAGACC and reverse AAGTGCATCATCGTTGTTCATACA (Accession No.: NM_031168); MUS TGF-β, forward TGATACGCCTGAGTGGCTGTCT and reverse CACAAGAGCAGTGAGCGCTGAA (Accession No.: NM_011577); and MUS NFKB, forward GCTGCCAAAGAAGGACACGACA and reverse GGCAGGCTATTGCTCATCACAG (Accession No.: NM_008689). MUS GAPDH was used as the reference gene, with the following primer pair: forward ACCCAGAAGACTGTGGATGG and reverse ACACATTGGGGGTAGGAACA (Accession No.: NM_008084). Multiple reactions were conducted in a 25 μL volume followed by cycling steps (40 cycles) to provide melting curves using RotorGene 6000 (QIAGEN, ABI System, USA). The 2-ΔΔCT method was applied to assess the gene expression, with each sample subjected to three separate amplifications.^70,71^

### 2.12 Assessment of the protein expression of apoptotic proteins

The western blot assay was employed to evaluate the expression levels of P53, cytochrome c, Bax, caspase-3, and Bcl-2 proteins. Protein extraction was performed using the ReadyPrep™ Protein Extraction Kit (Bio-Rad, Cat. #163-2086) according to the manufacturer's instructions. Protein concentrations were determined with the Bradford Protein Assay Kit (Bio Basic Inc., Cat. #SK3041), following the manufacturer's protocol. To ensure independent experimental replicates, western blot analysis was performed in three independent replicates, with protein extracted from three separate biological samples. For each replicate, electrophoresis, membrane transfer, and antibody incubations were carried out independently under identical conditions. For each protein sample replica, 20 μg of total protein from experimental and control groups was loaded onto the same SDS-PAGE gel. Samples were mixed with an equal volume of 2× Laemmli sample buffer containing 4% SDS, 10% 2-mercaptoethanol, 20% glycerol, 0.004% bromophenol blue, and 0.125 M Tris–HCl (pH 6.8). The mixture was heated at 95 °C for 5 minutes to ensure protein denaturation prior to electrophoresis. Each independent biological replicate was processed using a separate SDS-PAGE gel, with a fresh molecular weight ladder (Precision Plus Protein™ Dual Color Standards, Bio-Rad, Cat. #161-0374) loaded to confirm band size. Proteins were separated using SDS-PAGE with the TGX Stain-Free™ FastCast™ Acrylamide Kit (Bio-Rad, Cat. #161-0181) and subsequently transferred onto PVDF membranes using the BioRad Trans-Blot Turbo system. The transfer was carried out in 1× transfer buffer (25 mM Tris, 190 mM glycine, and 20% methanol) at 25 V for 7 minutes. To block non-specific binding, the membranes were incubated with TBST buffer (20 mM Tris, pH 7.5, 150 mM NaCl, 0.1% Tween 20) containing 3% bovine serum albumin (BSA) for 1 hour at room temperature. The membranes were then incubated overnight at 4 °C with primary antibodies diluted in TBST buffer according to the manufacturer's recommendations, including P53 (1 : 1000, Cell Signaling Technology, Cat. #9282, Massachusetts, USA), cytochrome c (1 : 1000, Proteintech, Cat. #66264-1-Ig, Planegg-Martinsried, Germany), Bax (1 : 500, Santa Cruz Biotechnology, Cat. #sc-7480, Heidelberg, Germany), caspase-3 (1 : 1000, Cell Signaling Technology, Cat. #9662, Massachusetts, USA), and Bcl-2 (1 : 500, Santa Cruz Biotechnology, Cat. #sc-7382, Heidelberg, Germany). Following incubation, the membranes were rinsed 3–5 times with TBST and subsequently incubated for 1 hour at room temperature with HRP-conjugated secondary antibodies (Goat anti-rabbit IgG, Novus Biologicals, Cat. #NBP1-74718, dilution 1 : 2000). The membranes were washed again 3–5 times with TBST and treated with Clarity™ Western ECL Substrate (Bio-Rad, Cat. #170-5060) according to the manufacturer's instructions. All experimental and control samples within a given replicate were run on the same gel/blot to ensure valid comparative analysis. The experiment for each target protein was repeated in three independent biological replicates, each using a separate SDS-PAGE gel to ensure reproducibility. Each blot was imaged separately using a CCD camera-based ChemiDoc MP imaging system, ensuring independent analysis of each replicate. Band intensities were quantified using ImageJ software and normalized against β-actin, a stable internal control.^72^

### 2.13 Histopathological study

Histopathological evaluations of the liver and kidney specimens from all the experimental groups were performed for the formalin and paraffin-embedded sections. After fixation of the tissues, sections were mounted on slides with a thickness of 5 μm and subsequently stained with hematoxylin and eosin (H&E). Microscopic examinations were conducted using a Nikon Eclipse E800 microscope equipped with a camera for image capture.^73–75^

### 2.14 Molecular docking study

The 3D crystal structures of proteins (a) TGF-β (PDB id: 1PY5),^76^ (b) glutathione transferase P1-1 (GSTP1-1, PDB id: 2 A2R),^77^ (c) superoxide dismutase-1 (SOD1, PDB id: 4A7U),^78^ (d) tumor protein P53 (PDB id: 1TUP),^79^ and (e) caspase-9 (PDB id: 1NW9)^80^ were downloaded from the ‘Protein database bank’ available online at https://www.rcsb.org/structure/. The two-dimensional (2D) structures of the native ligands and ‘BTHP’ were first sketched in ‘ChemDraw V.12.0’ and saved in ‘.mol’ files, which were further imported into either the ‘MOE V. 2015’ Platform or Autodock Vina software (https://vina.scripps.edu, the Scripps Research Institute's Laboratory of Molecular Graphics) and subsequently processed as per protocols available in the literature.^24,43,44,47,48,81–84^ For MOE platform, the ligand docking site was selected *via* the dummy atoms method, and a triangle matcher and London dG were used for placement and scoring. Employing stiffer receptors and GBVI/WSA dG, the optimal site was chosen based on favorable scores, binding modes, and RMSD values.^43,44,47,48,81,82,84^ The AutoDock utilized the PDBQT molecular structure file format (Trott and Olson, 2010), and the experiment was conducted 100 times and the protein–ligand complex with the highest binding affinity (kcal mol^−1^) was chosen.^83,85,86^

### 2.15 Statistical analysis

Quantitative parametric and nonparametric data are expressed as the mean ± standard deviation (SD). The results are expressed as the mean ± standard error (SEM). The data were tested for normality using the Shapiro–Wilk test before analysis. To compare multiple experimental groups, one-way ANOVA was utilized followed by Tukey's *post hoc* test. The percentage change was calculated for the treatment groups compared to the respective control group. A *p* value ≤0.05 was considered to indicate statistical significance (*p ≤ 0.05, **p ≤ 0.01, ***p ≤ 0.001, and ****p ≤ 0.0001). All the statistical analyses and graphical representations were conducted utilizing GraphPad Prism version 9.0.1 and SPSS version 25 software platforms. Appropriate statistical tests were selected based on the number of groups and the distribution of the data.

## 3. Results

### 3.1 Determination of the lethal dose (LD)

The role of LD determination studies in experimental animals is highly valued in drug discovery and development. To evaluate the safety of BTHP, various doses (1–100 mg kg^−1^, *n* = 3/group) were tested in experimental mice. At doses up to 50 mg kg^−1^, no adverse effects were observed, as evidenced by normal movement, activity, behavior, and excretion. However, at the 100 mg per kg dose, immediate adverse effects were noted, including reduced movement, lethargy, and signs of exhaustion. All mice in this dose group succumbed shortly thereafter. Based on these findings, 50 mg kg^−1^ was determined to be the maximum tolerated dose (MTD or LD0).

### 3.2 Assessment of the effective antiproliferative dose of BTHP

**Fig. 2:**
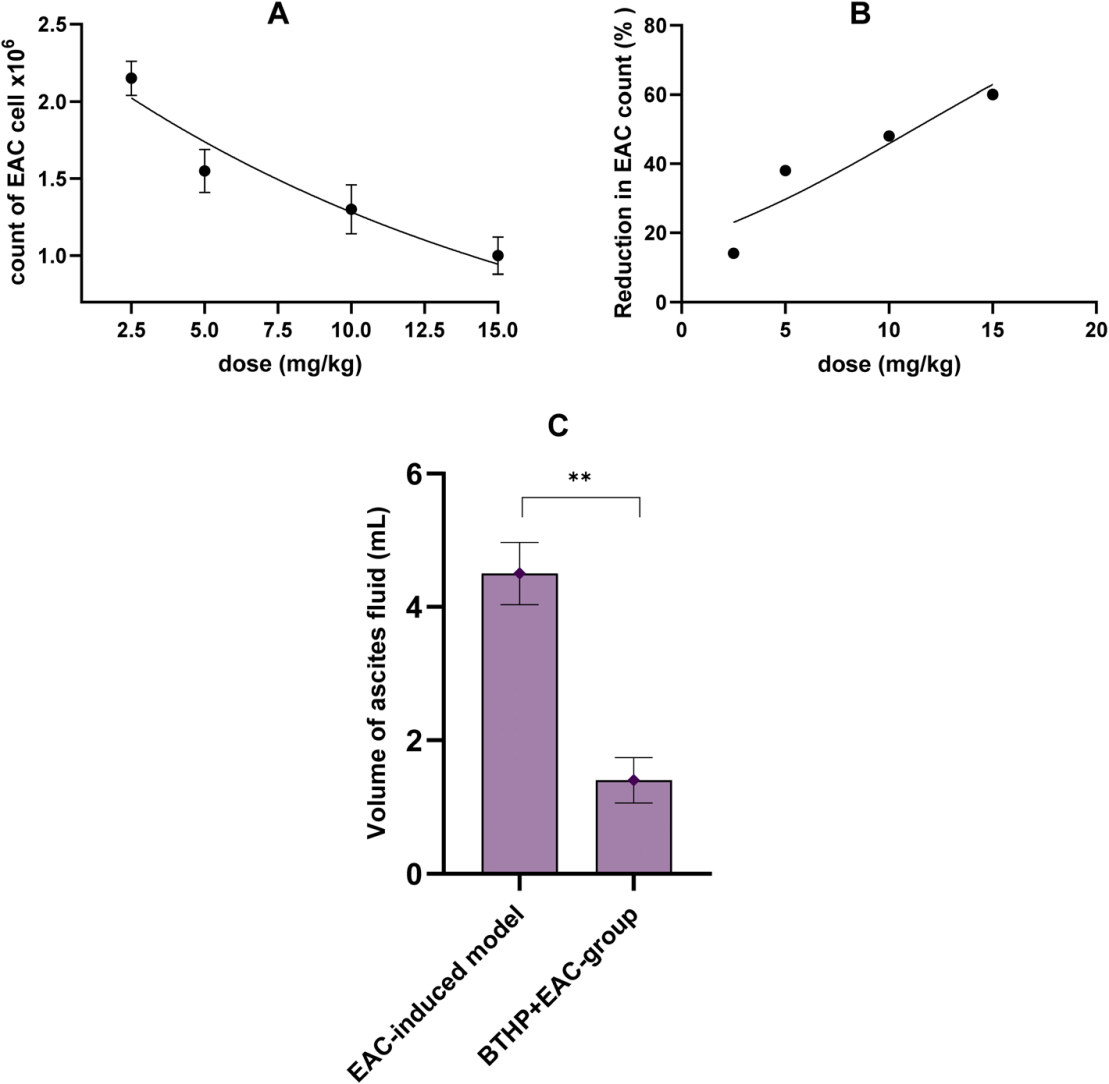
Effect of BTHP treatment on EAC proliferation in an EAC-induced mouse model. (A) The mean number of EAC cells after treatment with 2.5, 5, 10, or 15 mg per kg per day BTHP for 14 days. (B) The reduction (%) in the EAC cell count after treatment with 2.5, 5, 10, or 15 mg per kg per day BTHP for 14 days. (C) The impact of BTHP treatment at 5 mg per kg per day for 14 days on the volume of EAC cells. The data (*n* = 3, mean ± SEM) are considered significant at *p* ≤ 0.05 (****p ≤ 0.0001, ***p ≤ 0.001, **p ≤ 0.01, and *p ≤ 0.05).

The effective dose of BTHP required to substantially inhibit EAC cell proliferation in animal models was determined using a dose–response curve. In this regard, five groups of EAC-bearing mice were administered 2.5, 5, 10, or 15 mg per kg per day BTHP for 14 days, and subsequently, EAC cell proliferation was evaluated. As shown in Fig. 2, the administration of BTHP induced mitigation in the viability of EAC cells in a dose-dependent manner. Notably, the curve demonstrated pronounced inhibitory efficacy at the 5 mg per kg dosage, which was marked by a significant reduction in the EAC cell count (38%) and optimal effective dose for further testing in comparison to those of the EAC-induced model. Furthermore, the assessment of ascites fluid volume showed a significant (*p* ≤ 0.01) decrease in the volume of ascites fluid in the BTHP-treated EAC-induced model compared to that in the EAC-induced model (Fig. 2).

### 3.3 Effect of BTHP on lifespan prolongation and body weight

The prolongation of the lifespan of EAC-bearing mice is the main reliable standard for evaluating the efficiency of anticancer potential.^87,88^ Therefore, we evaluated the effect of BTHP administration (5 mg per kg per day) on life span prolongation in an EAC-induced mouse model. Our findings showed that the EAC-induced mice exhibited a mean survival duration of 16 days (day of first death = 15, day of last death = 17), while the BTHP-treated EAC-induced mice (5 mg per kg BW) displayed a marked extension of lifespan to a mean of 21 days (day of first death = 19, day of last death = 23), with an increase of 131.25% compared to that of the EAC-induced group. Furthermore, the ratio of EAC volume of the BTHP-treated group to that of the EAC-control group (*T*/*C* ratio) was 31.11%, indicating the potential anticancer activity of BTHP (Table 1).^63^ The assessment of body weight change indicated that the administration of EAC-cells induced a body weight change of 22%, while the BTHP-treated model revealed a body weight change of 8%, supporting the efficacy of BTHP in reducing the EAC volume.

**Table 1:** Effect of BTHP treatment on the body weight change and life span prolongation in the EAC-induced model (*n* = 6/group)

Parameters		Groups	
		EAC-induced model	BTHP-treated group (5 mg kg^−1^)
Mean of survival days		16	21
% Change (increase in life span)		—	131.25
*T*/*C* ratio (%)		—	31.11
Body weight (g)	Before	25 ± 0.64	25 ± 0.64
	After	30.5 ± 0.8	27 ± 0.5
Body weight change (%)		22%	8%

### 3.4 Effect of BTHP on the expression levels of antioxidant and oxidative stress biomarkers in the liver and kidney

**Fig. 3:**
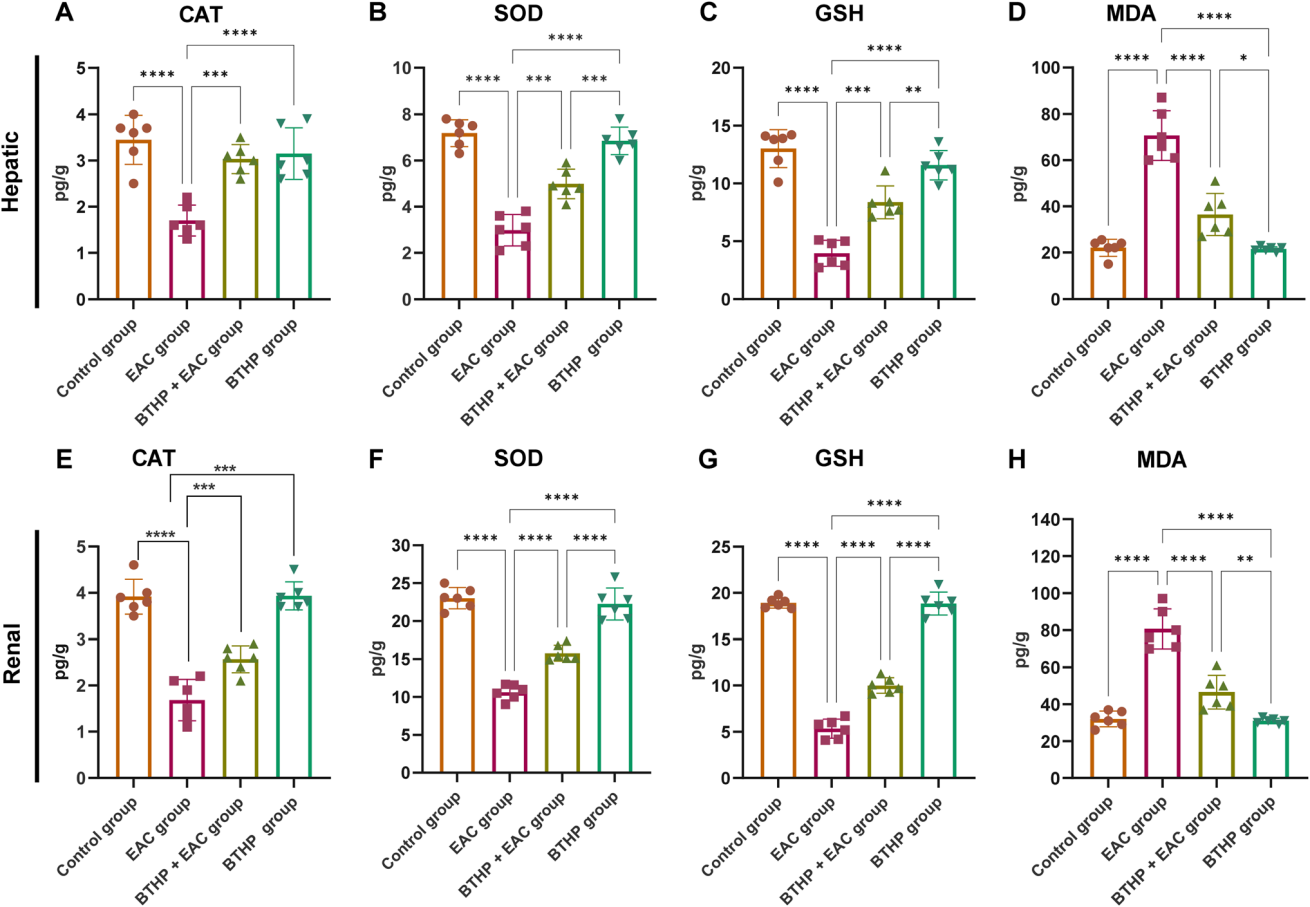
Effect of BTHP (5 mg per kg per day, 14 days) on the expression of different oxidative stress and antioxidant biomarkers in liver and kidney tissue of EAC-induced mice. (A–D) Effect of BTHP (5 mg per kg per day, 14 days) on the expression of hepatic catalase (A), SOD (B), GSH (C), and MDA (D) levels. (E–H) Effect of BTHP (5 mg per kg per day, 14 days) on the expression of renal (E), SOD (F), GSH (G), and MDA (H) levels. The data (*n* = 6, mean ± SEM) are considered significant at *p* ≤ 0.05 (****p ≤ 0.0001, ***p ≤ 0.001, **p ≤ 0.01, and *p ≤ 0.05).

Based on the positive effects of BTHP on EAC-induced mice, we sought to further assess and confirm the therapeutic potential of BTHP by examining changes in different biochemical and histological parameters. Initially, we aimed to assess whether the antitumor activity of BTHP is associated with its ability to modulate oxidative stress in an EAC-induced model. In this regard, we explored the expression levels of different antioxidant and oxidative stress biomarkers, including SOD, GSH, catalase, and MDA among the experimental groups. As shown in Fig. 3, compared with those in the control group, the expression levels of antioxidant biomarkers (catalase, SOD, and GSH) in the hepatic homogenates of the EAC-induced group were significantly attenuated (*p* ≤ 0.0001), while the level of lipid peroxidation (MDA) was substantially upregulated (*p* ≤ 0.0001). Compared with those in the control group, the expression levels of hepatic SOD, GSH, catalase, and MDA in the control group treated with BTHP alone for 14 days (5 mg per kg per day) were not significantly different. However, the administration of BTHP (5 mg per kg per day, 14 days) to the EAC-induced group substantially mitigated the hepatic oxidative stress biomarker MDA levels (*p* ≤ 0.0001) while significantly augmented the levels (*p* ≤ 0.001) of different antioxidant biomarkers (catalase, SOD, GSH) as compared to those in the EAC-treated group. A similar trend was observed in the renal homogenate. Compared with those in the control group, the renal MDA levels in the EAC-treated group were significantly (*p* ≤ 0.0001) elevated, while the SOD, GSH, and catalase levels were mitigated (*p* ≤ 0.0001). The expression levels of the examined renal oxidative stress and antioxidant markers were not significantly different between the control group treated with BTHP (5 mg per kg per day, 14 days) and the control group. However, compared with those in the EAC-induced model, the MDA levels in the BTHP-treated EAC model (5 mg per kg per day, 14 days) significantly increased SOD (*p* ≤ 0.0001), GSH (*p* ≤ 0.0001), and catalase (*p* ≤ 0.001) levels but significantly (*p* ≤ 0.0001) decreased.

### 3.5 Effect of BTHP on the serum biochemical markers of liver and kidney function

**Fig. 4:**
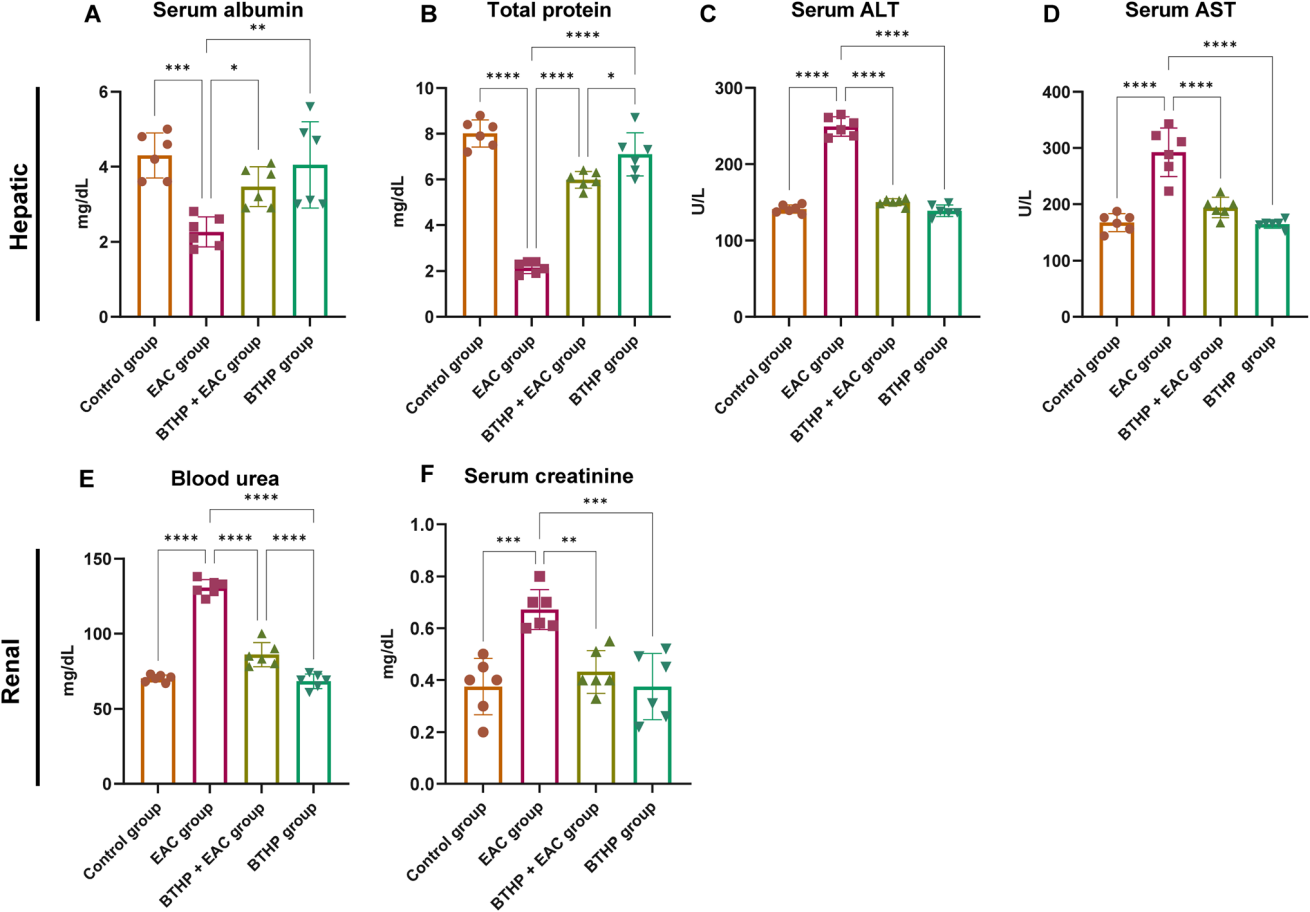
Effect of BTHP (5 mg per kg per day, 14 days) on liver and renal function in the EAC-rat models. (A–D) Effect of BTHP (5 mg per kg per day, 14 days) on the expression of serum liver biomarkers ALB (A), total protein (B), ALT (C), and AST (D) levels. (E and F) Effect of BTHP (5 mg per kg per day, 14 days) on the expression of serum renal biomarkers creatinine (E) and blood urea (F) levels. The data (*n* = 6, mean ± SEM) are considered significant at *p* ≤ 0.05 (****p ≤ 0.0001, ***p ≤ 0.001, **p ≤ 0.01, and *p ≤ 0.05).

Next, we explored the hepatorenal protective effects of BTHP administration on the EAC-induced model by assessing the expression levels of different serum biochemical markers associated with liver and kidney function among the experimental groups. As shown in Fig. 4, compared with those in the control group, the levels of blood urea and serum creatinine in the EAC-induced group were significantly elevated by 0.76 and 130.8 mg dL^−1^, respectively. These results indicate that EAC treatment substantially induced kidney dysfunction. Alternatively, treatment of the control group with BTHP (5 mg per kg per day, 14 days) had no significant effect on renal functional biomarkers compared to those in the untreated control group. Interestingly, compared with those in the EAC-induced group, the levels of creatinine (*p* ≤ 0.01) and blood urea (*p* ≤ 0.0001) in the BTHP-treated EAC-induced group were substantially mitigated (0.43 and 86 mg dL^−1^, respectively), suggesting improvements in kidney function. Similarly, the assessment of the serum liver function parameters in the EAC-induced group revealed a substantial elevation in the levels of ALT and AST (249.3 and 292.3 U L^−1^, respectively; *p* ≤ 0.0001), but also a significant reduction in the total protein and albumin levels (2.12 and 2.26 mg dL^−1^, respectively; *p* ≤ 0.0001) as compared to those in the control group. BTHP treatment in the control group had no significant effect on the assayed biochemical markers of liver function, as they maintained normal levels. However, the administration of BTHP (5 mg per kg per day, 14 days) to the EAC-induced group significantly improved liver and kidney functions, as indicated by significant decreases in the serum ALT and AST levels and significant increases in the serum ALB and total protein levels compared to those in the EAC-induced group. As shown in Fig. 4, BTHP treatment substantially mitigated the levels of the liver function biomarkers ALT and AST (150 U L^−1^ and 194.1 U L^−1^, respectively; *p* ≤ 0.0001) but also significantly augmented the total protein and albumin levels (5.98 (*p* ≤ 0.0001) and 3.46 g dL^−1^ (*p* ≤ 0.01), respectively) as compared to those in the EAC-induced group. These findings indicate that BTHP treatment significantly improved kidney and liver functions in the EAC-treated group, as indicated by decreased ALT, AST, blood urea, and creatinine levels and improved total protein and albumin levels.

### 3.6 Effect of BTHP on the expression levels of proinflammatory markers in the liver and kidney

**Fig. 5:**
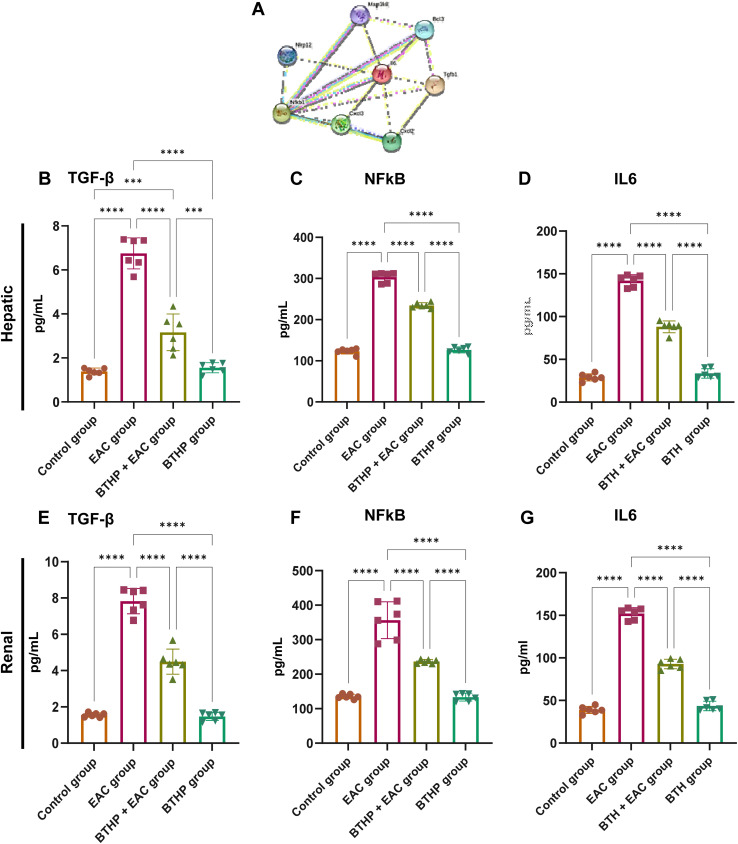
Effect of BTHP (5 mg per kg per day, 14 days) on the expression of various inflammatory biomarkers in the liver and kidney of EAC-mouse models. (A) A typical association network in STRING for computational protein interaction analysis of studied targets. The colored nodes denote the proteins, while the edges signify the protein–protein associations. The interacting nodes with the central hub IL6 represented the predicted functional protein partners, which included “NF-kβ 1” nuclear factor NF-kappa-B p105 subunit, ‘TGFB transforming growth factor beta-1 proprotein, “Cxcl3” C–X–C motif chemokine 3, “Cxcl2” C–X–C motif chemokine 2, “Bcl3” B-cell lymphoma 3 protein homolog, ‘Nlrp12’ NACHT, LRR and PYD, domain-containing protein 12, ‘and Map3k8’ mitogen-activated protein kinase 8 https://version-12-0.string-db.org/cgi/network?networkId=bA0MMxvoAPWZ. (B–D) Effect of BTHP (5 mg per kg per day, 14 days) on hepatic TGF-β (B), NF-κB (C), and IL6 (D) tissue expression. (E–G) Effect of BTHP (5 mg per kg per day, 14 days) on renal TGF-β (E), NF-κB (F), and IL6 (G) tissue expression. The data (*n* = 6, mean ± SEM) are considered significant at *p* ≤ 0.05 (****p ≤ 0.0001, ***p ≤ 0.001, **p ≤ 0.01, and *p ≤ 0.05).

To examine whether the BTHP antitumor activity is associated with its capability to target the inflammatory pathway in the EAC-treated group, we aimed to assess the expression levels of a set of proinflammatory markers in liver and kidney tissues. In this regard, we constructed a protein–protein interaction network for a set of proinflammatory targets utilizing the STRING database (version 12.0). The overall protein–protein interaction enrichment (PPI) *p*-value was 0.000858. As illustrated in Fig. 5 and Table S2,† IL-6 interacted with several proteins, including Cxcl3, Cxcl2, Bcl3, Nlrp12, and Map3k8, with strong confidence levels. Furthermore, NF-κB directly interacted with IL-6, with a confidence score of 0.985, and with TGF-β, with a confidence score of 0.892. Based on these findings, we examined the levels of NF-κB, TGF-β, and IL6 genes in liver and kidney tissues from the experimental groups. Our investigations revealed that compared with those in the control group, the hepatic and renal TGF-β, NF-kβ, and IL6 levels in the EAC-treated group substantially elevated.

Furthermore, administration of BTHP to the control group had a nonsignificant influence on the examined hepatic and renal proinflammatory cytokines compared to those in the control group. However, treatment of the EAC-induced model with BTHP (5 mg per kg per day, 14 days) substantially (*p* ≤ 0.0001) attenuated the levels of TGF-β, NF-κB, and IL6 genes in liver and kidney tissues as compared to those in the EAC-treated group. As indicated in Fig. 5, treatment of EAC-treated mice with BTHP significantly reduced the levels of hepatic TGF-β, NF-kβ, and IL-6 from 6.75, 303.35, and 142 pg mL^−1^, respectively, in the EAC model to 3.16, 234.56 and 88 pg mL^−1^, respectively, in the BTHP-treated EAC-induced group. TGF-β, NF-kβ, and IL-6 had similar effects on kidney tissues, as the levels of these proinflammatory cytokines in the EAC model were increased by 7.82, 356.5, and 152 pg mL^−1^, respectively. Significantly attenuated levels of renal TGF-β, NF-kβ, and IL-6 were also detected in the BTHP-treated EAC-induced group (4.49, 236.86, and 92.8 pg mL^−1^, respectively).

### 3.7 Effect of BTHP on the expression levels of apoptotic markers

**Fig. 6:**
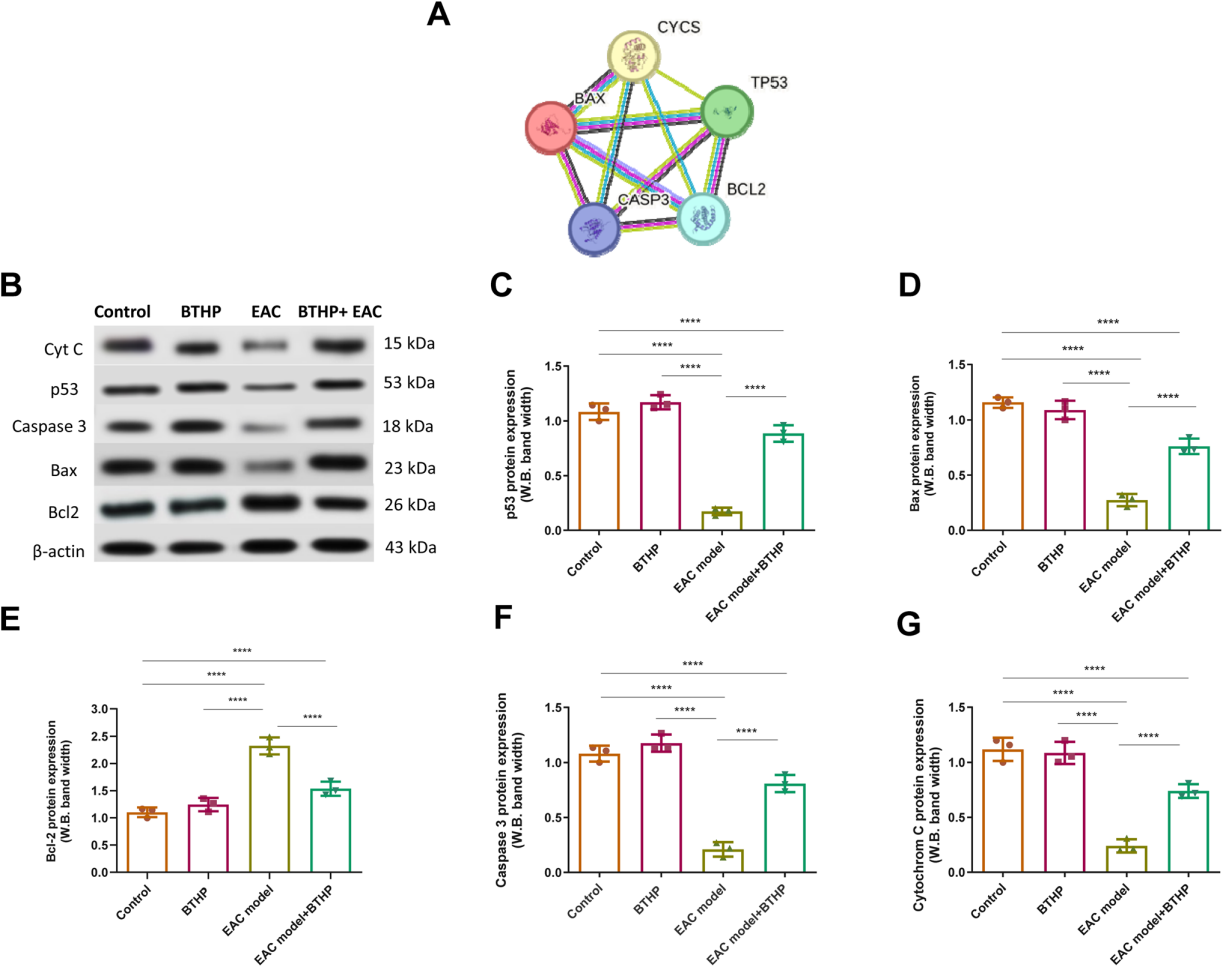
Effect of BTHP treatment (5 mg per kg per day, 14 days) on the expression of various serum apoptotic proteins in the EAC-mouse models. (A) A typical association network in STRING for computational protein–protein interaction analysis of studied apoptotic targets. The colored nodes denote the proteins, while the edges signify the protein–protein associations between p53 ‘TP53’, apoptosis regulator BAX “BAX”, ‘cytochrome c, “CYCS”, caspase-3 subunit p12 “CASP3”, and apoptosis regulator Bcl-2, ‘BCL2’ (Table S3†) https://version-12-0.string-db.org/cgi/network?taskId=bL11f6y0nNPa&sessionId=bFOZWno1LXYK. (B–G) Effect of BTHP treatment (5 mg per kg per day, 14 days) on the protein expression levels of apoptotic proteins (B) including serum p53 (C), BAX (D), Bcl-2 (E), caspase 3 (F), and cytochrome C (G). The data (*n* = 3, mean ± SEM) are considered significant at *p* ≤ 0.05 (****p ≤ 0.0001, ***p ≤ 0.001, **p ≤ 0.01, and *p ≤ 0.05).

To explore whether the anticancer activity of BTHP is correlated with its ability to induce apoptotic pathways in the EAC model, we envisioned examining the expression of serum apoptotic markers. Toward this, we initially performed computational protein–protein interaction analysis of a set of apoptotic targets, including p53, BAX, Bcl-2, caspase 3, and cytochrome C utilizing the STRING database (version 12.0). The computational analysis revealed a PPI enrichment *p*-value of 0.0156, supporting a significant protein–protein interaction between the selected targets. As indicated in Fig. 6 and Table S3,† the selected targets showed a significant interaction with each other with strong confidence scores ranging from 0.884–0.999. Encouraged by these results, we aimed to examine the expression levels of p53, BAX, Bcl-2, caspase 3, and cytochrome C proteins utilizing western blot analysis among experimental groups. As indicated in Fig. 6, the EAC-treated group displayed a significant (*p* ≤ 0.0001) attenuation in the expression levels of p53, BAX, caspase 3, and cytochrome C proteins, while a considerable (*p* ≤ 0.0001) elevation was reported for Bcl-2 protein, as compared to the control group. Interestingly, treatment of the control group with BTHP demonstrated a non-significant impact on the expression levels of the examined apoptotic proteins, as compared to the control model. On the other hand, the administration of BTHP (5 mg per kg per day, 14 days) to the EAC-treated group significantly (*p* ≤ 0.0001) elevated the levels of p53, BAX, caspase 3, and cytochrome C levels, but also substantially (*p* ≤ 0.0001) attenuated the expression of Bcl-2 protein, as compared to EAC-induced model. These results indicate that the anticancer potential of BTHP could be associated with its ability to induce apoptosis in the EAC-induced model by modulating the key regulatory proteins including p53 and their transcriptional targets (Bax and Bcl-2), but also the cytochrome C and caspase 3 proteins.

### 3.8 Histological examination of the effect of BTHP on kidney and liver tissues

**Fig. 7:**
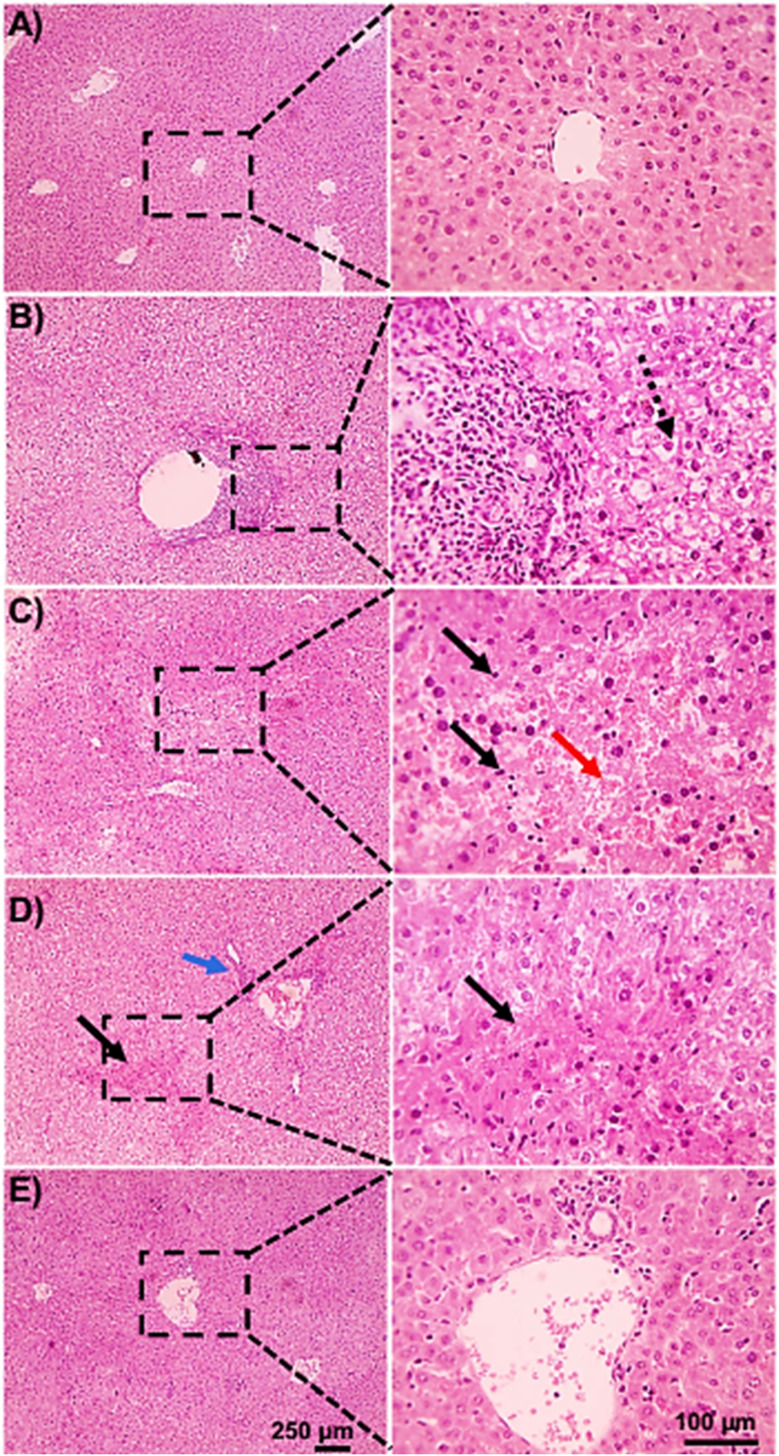
Microscopic analysis of H&E-stained liver sections of the control group (A), EAC group (B–D), and BTHP + EAC group (E). (A) Control group showing a normal hepatic architecture with radiating hepatic cords, normal central vein, portal areas, and sinusoids. (B–D) Liver sections from the EAC group showing massive aggregation of EAC cells admixed with mononuclear cells in portal areas (black arrowheads), diffuse hepatocytes hydropic degeneration (dashed arrows), hemorrhage (red arrows) (B and C), multifocal areas of hepatocytes coagulative necrosis (black arrows) (D). Portal fibrosis and inflammation (blue arrows). (E) Liver sections of the BTHP + EAC group showed much fewer mononuclear cellular infiltration (black arrowheads) with the restoration of hepatic tissue architecture.

Based on the biochemical findings, we aimed to further confirm the hepatorenal protective effects of BTHP on the EAC-induced model by conducting detailed microscopic histological assessments of H&E-stained liver and kidney tissues. As shown in Fig. 7A, the liver tissues of the control group displayed a normal hepatic architecture with hexagonal hepatic lobules, hepatocyte plates radiating from the central vein, normal portal areas, and sinusoids. However, liver histopathological examination of the EAC-treated group revealed inflammation indicated by mononuclear cellular infiltration in some portal areas, as well as fibrosis in other portal regions, suggesting the development of chronic pathological changes and tissue remodeling in response to EAC cell invasion, with the presence of diffuse hepatocytes hydropic degeneration Fig. 7B. Furthermore, hemorrhage and multifocal areas of hepatocyte coagulative necrosis were observed (Fig. 7C and D). Interestingly, the assessment of the liver tissues of the BTHP-treated EAC-induced group demonstrated a marked reduction in cellular infiltrates, hydropic degeneration, and necrosis of liver tissues (Fig. 7E).

**Fig. 8:**
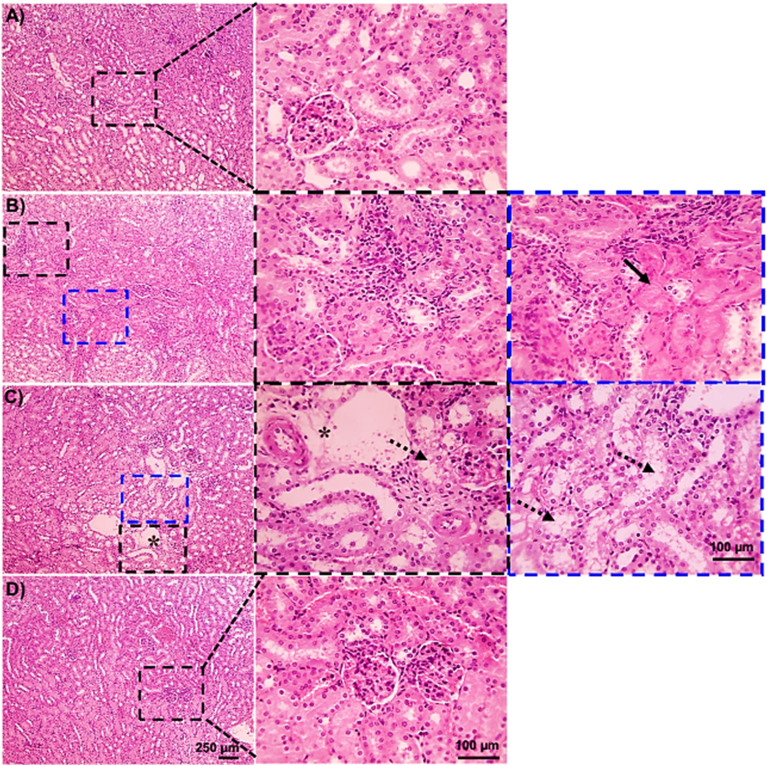
Microscopic analysis of H&E-stained kidney sections of the control group (A), EAC group (B and C), and BTHP + EAC group (D). (A) Control group showing normal glomeruli, tubules, and interstitial tissue. (B and C) Kidney sections from the EAC group showed massive aggregation of EAC cells admixed with mononuclear cells in perivascular tissue (black arrowheads) accompanied by tubular coagulative necrosis (black arrows) in some sections (B), with perivascular edema (*) and tubular epithelium hydropic degeneration (dashed arrows) in other sections (C). (D) Kidney sections of BTHP + EAC group showed improved renal tissue histology.

Next, we examined H&E-stained kidney sections from the experimental groups utilizing a microscopic analysis. As shown in Fig. 8A, the control group exhibited normal renal tissue structures including normal glomeruli, tubules, and interstitial tissues. Alternatively, the EAC-induced group indicated massive aggregation of EAC cells with severe renal tissue damage, including massive aggregation of mononuclear cells in perivascular tissue with tubular coagulative necrosis in some sections (Fig. 8B), with the presence of perivascular edema and tubular epithelium hydropic degeneration in tubular epithelial cells in other sections (Fig. 8C). Histopathological examination of renal tissue from the BTHP-treated mice in the EAC group revealed significant histological improvement, with mitigation in the number of mononuclear cells and less degenerated tubules, and the cells appeared more intact. Therefore, BTHP treatment effectively mitigated EAC-induced renal damage, helping to preserve the structural and functional integrity of the kidney (Fig. 8D).

### 3.9 Molecular modeling assessment

To further explore and affirm the antitumor mode of action of BTHP, we conducted extensive molecular docking studies of BTHP toward a set of key target proteins, including TGF-beta, glutathione transferase, superoxide dismutase, tumor protein P53, and caspase-9 proteins.

#### 3.9.1 Targeting the TGF-β type I receptor kinase domain (TbetaR-I)

**Fig. 9:**
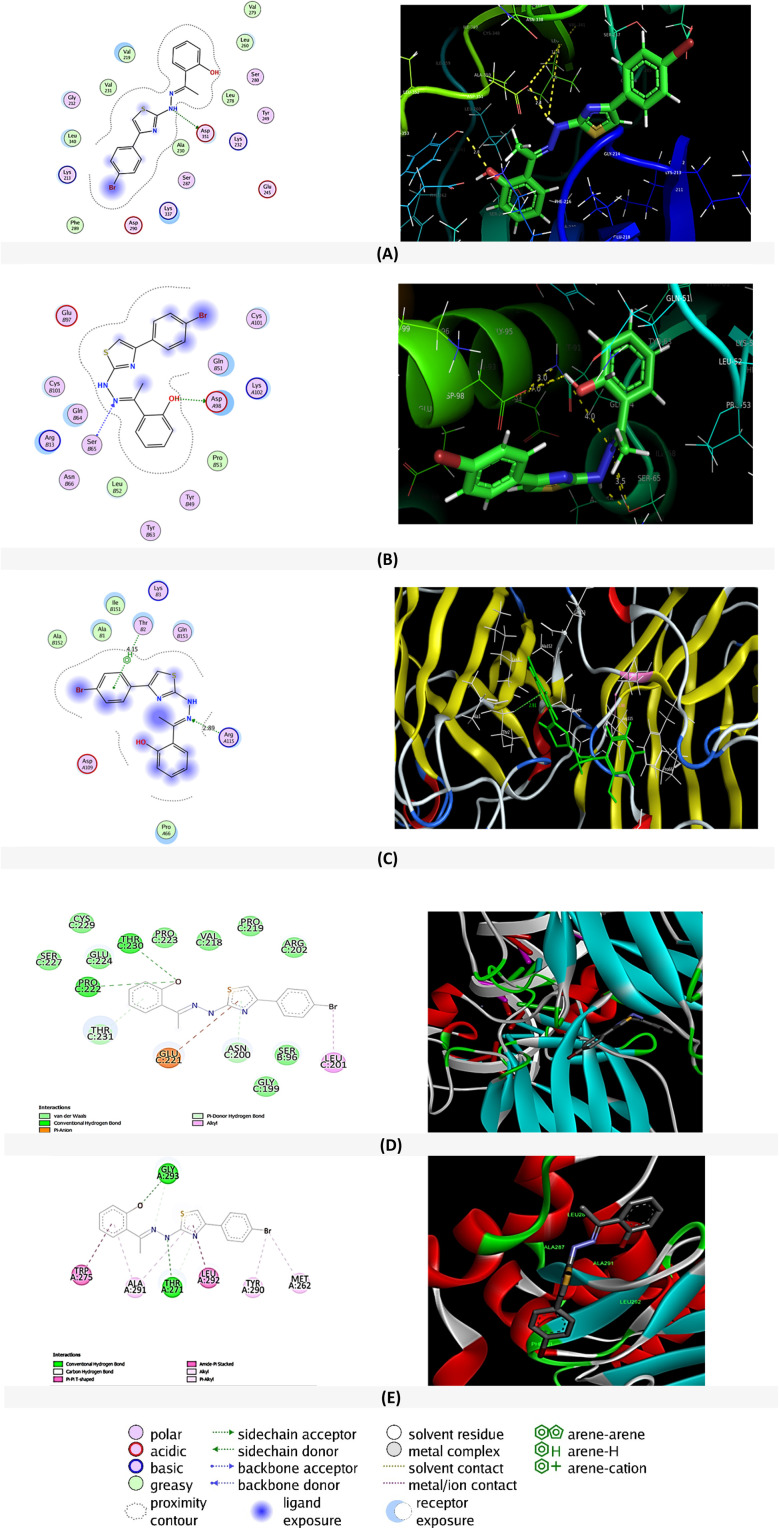
The 2D and 3D binding interactions of ‘BTHP’ toward (A) Tbeta receptor 1 kinase; (B) glutathione transferase P1-1 (GSTP1-1); (C) superoxide dismutase-1 (SOD1); (D) P53, and (E) caspase 9.

Members of the transforming growth factor-β (TGF-β) superfamily, which includes TGF-β1, TGF-β2, TGF-β3, activins, inhibins, and bone morphogenetic proteins (BMPs), communicate *via* transmembrane serine/threonine kinase receptors.^76^ These receptors are categorized into type I or ALK receptors and type II receptors. When TGF-β1 binds to type II receptors, it triggers the phosphorylation of type I receptors.^76^ Considering the importance of these receptors, including ‘TbetaR-I’, we carried out a molecular simulation for BTHP toward the binding pocket of the receptor (PDB id: 1PY5; resolution: 2.30 Å; organism(s): *Homo sapiens*^76^) along with the cocrystallized ligand ‘PY1 (4-(3-pyridin-2-yl-1*H*-pyrazol-4-yl)quinoline)’ (Fig. 9 and S3†). Upon docking, we observed that ‘BTHP’ displays a substantial binding affinity (docking score: −7.30 kcal mol^−1^) toward the target protein compared to the native ligand ‘PY1’ (docking score: −6.28 kcal mol^−1^). Furthermore, BTHP interacted with a set of amino acid residues, such as Asp 351, *via* a side chain acceptor (–NH – Asp351, distance: 2.4 Å, H-bonding) (Fig. 9). Other polar residues inside the binding pocket were also incorporated, including Gly212, Ser280, Tyr249, Glu245, and Ser 287. On the other hand, the cocrystallized ligand ‘PY1’ showed only two arene–H interactions with amino acid residues (1*H*-pyrazole – Val219 and pyridine – Val231) (Fig. S4†).

#### 3.9.2 Targeting the glutathione transferase pi

Nitric oxide (NO) plays key roles in various physiological processes and is often stabilized by molecules such as *S*-nitrosoglutathione (GSNO) that binds to glutathione transferases, enzymes that detoxify cells and are responsible for the transfer of several mutagenic and carcinogenic compounds.^89,90^ Glutathione transferase P1-1 (GSTP1-1), also known as e-GST in erythrocytes, is an enzyme found abundantly in mammals that detoxifies cells using GSH. Studies suggest that GSTP1-1 could serve as a valuable biomarker for detecting blood toxicity in kidney disease patients, as its overexpression in erythrocytes correlates with toxin levels in the body.^89^ To explore the interaction of ‘BTHP’ with GSHP1, we carried out docking simulations (PDB id: 2A2R; resolution: 1.40 Å; *R*-value free: 0.213).^91^ Our results showed that the compound ‘BTHP’ exhibited a greater binding affinity (docking score: −6.46 kcal mol^−1^) than did the bound native cocrystallized ligand ‘MES’ [2-(*N*-morpholino)ethanesulfonic acid] (docking score: −4.41 kcal mol^−1^) (Fig. 9 and S3†). The ligand ‘BTHP’ demonstrated two interactions: one with a backbone acceptor-type interaction with SerB65 (3.5 Å) and another with H-bonding with AspA98 (a side chain acceptor, 3.0 Å) (Fig. 9). MES demonstrated two side chain acceptor-type interactions with the amino acid residues TyrB108 and AsnB204 (Fig. S4†). Within the binding pocket, we observed a majority of polar residues, including CysA101, GlnB51, AspA98, TyrB49, TyrB63, SerB65, TyrB108, and AsnB204 (Table 2).

**Table 2:** The binding affinity and interaction of BTHP and cocrystallized ligands toward the various studied targets^a^

Target	Ligand	Score (kcal mol^−1^)	Interactions/residues	
			Hydrophilic	Hydrophobic
TGF-β	BTHP	−7.30	Asp351 (2.4 Å), Ser280, Tyr249, Lys232, Lys213	Val219, Val231, Leu340, Ala230, Phe289
	PY1	−6.28	Tyr249, Lys232, Lys213, Asp281, His283	Leu260, Ala230, Ala350, Val231, Val219, Val279, Leu340
GSTP1-1	BTHP	−6.46	AspA98 (3.0 Å), GlnB51, LysA102, CysA101, TyrB49, Ser65 (3.5 Å), Glu97, Gln64	ProB53, LeuB52
	MES	−4.41	TyrB108, AsnB204	IleB104, LeuB52, IleB203
SOD1	BTHP	−6.84	LysB3, ThrB2 (4.15 Å), GlnB153, Arg115 (2.89 Å), AspA109	IleB151, AlaB1, AlaB152, ProA66
	ALE	−4.75	Thr113, Ser107, Thr2, Cys111	Leu106 (2.89 Å), Ala1, Ile151
P53	BTHP	−7.60	Thr230, Thr231, Asn200, Ser96, Arg202, Pro222, Glu221, Leu201	Pro219, Cys229, Pro223, Gly199
CASP-9	BTHP	−8.9	Thr271, Gly293	Trp275, Ala291, Leu292, Tyr290, Met262

a TGF-β: transforming growth factor beta; GSTP1-1: glutathione *S*-transferase pi 1; SOD1: superoxide dismutase-1; P53: tumor protein P53; CASP-9: caspase 9; PY1: (4-(3-pyridin-2-yl-1*H*-pyrazol-4-yl)quinoline); MES: [2-(*N*-morpholino)ethanesulfonic acid]; ALE: l-epinephrine.

#### 3.9.3 Targeting superoxide dismutase-1 (SOD1)

SOD1, which binds copper and zinc ions, is one of three superoxide dismutases responsible for neutralizing free superoxide radicals and plays an important role in modulating oxidative stress.^78,92^ It acts as a homodimer in the cytoplasm and mitochondrial intermembrane space, converting superoxide radicals into oxygen and hydrogen peroxide. SOD1 is considered a potential therapeutic target due to its ability to modulate inflammation and malignant progression.^92^ In this regard, we investigated the ability of BTHP to target SOD1 by assessing its binding affinity toward the active site of SOD1. As shown in Table 2, our results revealed that BTHP exhibits a substantial binding affinity toward the active site (PDB id: 4A7U; resolution: 0.98 Å (ref. 78)), with a docking score of 6.84 kcal mol^−1^, compared to that of the original cocrystallized ligand ‘ALE’, with a docking score of −4.75 kcal mol^−1^. BTHP showed one arene–H-type interaction with ThrB2 (4.15 Å), together with one side chain donor interaction with ArgA115 – –NH–N= (distance: 2.89 Å) (Fig. 9). The native ALE exhibited one H-bonding interaction with the amino acid residue Leu106 (distance: 2.89 Å) (Fig. S4†). The polar residues found inside the pocket were LysB3, ThrB2, GlnB153, Arg115, and AspA109.

#### 3.9.4 Targeting the tumor protein p53

The tumor protein p53 is a key focus in cancer research as it plays a central role in maintaining genomic integrity. When DNA damage is detected, the p53 protein promotes apoptosis by inducing Fas expression through specific promoters and activation of TNF to prevent the propagation of damaged cells. Given the ability of BTHP to trigger apoptosis in breast cancer cells,^24^ we aimed to explore whether the apoptotic effect of BTHP is associated with its ability to target p53 protein.^93^ Toward this, we conducted a molecular docking study toward p53 protein (PDB id: 1TUP) and assessed the binding affinity score of BTHP. As shown in Fig. 9, the molecular modelling analysis indicated that BTHP exhibits a considerable binding affinity toward p53 protein with a score of −7.6 kcal mol^−1^. The BTHP showed the ability to bind to a set of amino acid residues including Thr230, Pro222, Glu221, and Leu201 residues. The binding was further established by the hydrophobic interactions with Pro219, Cys229, Pro223, and Gly199 residues (Table 2).

#### 3.9.5 Targeting the caspase-9

Caspase 9 is a key enzyme in the intrinsic pathway of apoptosis in response to mitochondrial signals. In cancers, mutations or dysregulation of caspase 9 can lead the cancer cells to survive despite DNA damage which contributes to tumor growth and resistance to treatments.^94^ To explore whether the apoptotic activity of BTHP is correlated to its ability to target caspase 9, we conducted a molecular modelling study toward caspase-9 (PDB id: 1NW9) (Fig. 9). Our analysis revealed that BTHP displays a substantial binding affinity toward caspase-9 (docking score: −8.9 kcal mol^−1^). As indicated in Table 2, BTHP interacted significantly with two main amino acid residues, mainly Gly293 and Thr271. Additionally, the binding was further supported by a network of hydrophobic interactions with Tyr262, Met262, Leu292, Ala291, and Trp275 residues.

Taken together, our docking simulation analysis indicated that BTHP exhibits considerable binding affinity and docking scores compared to those of the cocrystal ligands/s enclosed in the binding pockets of the studied proteins. Most interactions of ‘BTHP’ with the binding pocket of the examined proteins involved H-bonding, which contributed to the observed high binding scores. Other hydrophobic interactions were also observed within the binding pockets, which could also be associated with the binding pose and score of BTHP. In agreement with our biochemical analysis, the molecular modeling study confirmed the substantial affinity of BTHP for the SOD protein, supporting the association of this target protein with the pronounced antitumor activity of BTHP. Furthermore, our findings revealed that BTHP exhibits considerable binding affinities toward glutathione transferase and TbetaR1, supporting our findings that indicate the ability of BTHP to modulate GSH and TGF-β levels in the EAC-induced model. Our computational analysis further highlighted the possibility that BTHP targets several critical proteins including P53, and caspase-9 proteins, which could be associated with its pronounced apoptotic, antiproliferative and antitumor activities. Future research should be directed to explore the potential activity of BTHP toward these target proteins and to determine their correlation with the antitumor activity and mode of action of this compound.

## 4. Discussion

The novel synthetic 1,3-thiazole analog, BTHP, exhibited potential antiproliferative activity toward breast cancer cells.^24^ In the present study, we extended our investigations to explore the anticancer potential of BTHP in EAC female Swiss albino mice. The EAC model exhibits several advantages including a high tumor take rate, rapid growth, and facile development. This model has been widely recognized for its similarity to human hormone-positive breast cancer, requiring the estrogen and progesterone-rich hormonal environment found in females for successful tumor establishment and growth. Male mice are unable to support the growth of Ehrlich ascites carcinoma due to the absence of the requisite hormonal milieu. Therefore, adult female Swiss albino mice were selected for this study to ensure the validity and translational relevance of the findings to hormone-sensitive breast cancer models in humans.^95,96^ Unlike complex models, the EAC model can be used in immunocompetent mice, facilitating the study of tumor–immune interactions. When it comes to the assessment of anticancer effects, the EAC model can be extrapolated to other breast cancer models by providing insights into molecular mechanisms, dosage, toxicity, and immune modulation.^95,97^ The LD determination in experimental animals is highly valued in drug discovery and development.^98–100^ The safety profile of BTHP was assessed during dose optimization. No adverse effects were observed in mice at doses up to 50 mg kg^−1^, indicating good tolerability. However, at 100 mg kg^−1^, the compound caused immediate behavioral changes, including reduced activity and lethargy, followed by mortality in all treated animals. These findings emphasize the importance of dose selection, and our choice of 5 mg kg^−1^ as the effective dose ensures safety while achieving therapeutic efficacy. On the other hand, previous reports revealed that doses up to 500 mg kg^−1^ were safe for thiazoles, with no observed mortalities.^101^ The present study displayed that the administration of BTHP at 5 mg kg^−1^ is an effective dosage-induced mitigation in the viability and volume of EAC cells. Although BTHP exhibited a dose-dependent inhibition of EAC viability, the 5 mg per kg BW dose was selected as the minimal effective dose to ensure minimal risk of toxicity and to avoid adverse effects of overdose. The prolongation of the lifespan of EAC-bearing mice is the main reliable standard for evaluating the efficiency of anticancer potential.^87,88^ Our findings showed that the BTHP-treated EAC-induced mice displayed a marked extension of lifespan compared to that of the EAC-induced group. Similar effects have been previously reported for a wide variety of thiazoles, such as 5-(pchlorophenyl)-2-(ethoxy carbonylmethylidene)hydrazine-1,3-thiazole (CP1)^101^ and 3-(2-(2-(10-chloroanthracen-9-yl)-methylidene)hydrazino)thiazol-4-yl)-2*H*-chromen-2-one (5r).^102^ The potential for nitrosamine formation in pharmaceuticals is an important safety consideration. While the 1,3-thiazole scaffold is part of our compound, nitrosamine formation generally requires the presence of secondary or tertiary amine groups and nitrosating agents. Our compound does not contain nitrosatable amine functionalities, and a preliminary evaluation of the synthetic route confirmed the absence of nitrosamine precursors. Thus, the risk of nitrosamine formation for this compound is considered minimal, consistent with published guidelines and literature on nitrosamine risk assessment.^103^

The administration of EAC tumor cells induced an oxidative stress involved in tumor pathogenesis. Lipid peroxidation promotes tumor proliferation by enhancing genomic instability and stimulating cytokines to induce proinflammatory signals which provides an inflammatory microenvironment to tumor cells as a source of essential nutrients, such as excessive oxidized lipids.^104,105^ The accumulation of MDA, a lipid peroxidation marker, leads to the inhibition of cancer immune responses by impairing the anticancer ability of natural killer cells and enhancing T lymphocyte dysfunction.^105–107^ This evidence may explain the elevated levels of MDA in the EAC-treated group. The antioxidant defense mechanisms provided by SODs are essential for manipulating oxidative stress. Nevertheless, SOD is often reduced or absent in cancer cells, which is consistent with the findings of the present study.^108^ Therefore, the upregulation of SOD has been suggested as a targeted cancer therapy for ameliorating tumor proliferation.^109,110^ One of the key cellular resistance strategies against oxidative stress is the upregulation of catalases that are found in liver and kidney tissues. Several studies have reported a reduction in catalase activity in cancer cells, as observed in the present study in an EAC-treated model.^111,112^ GSH is a thiol-based antioxidant that plays an important role in maintaining the cellular redox balance of the essential thiol status of proteins. Similarly, GSH deficiency leads to increased vulnerability to oxidative stress, which is essential for tumor progression and metastasis.^113,114^ The current study revealed that the BTHP administration mitigated the expression of the hepatic and renal oxidative stress biomarker MDA while significantly elevating the expression of different antioxidant biomarkers in both tissues (catalase, SOD, GSH) compared to those in the control EAC-treated group. These findings reveal that the antitumor activity of BTHP could be linked to its capability to manipulate the antioxidative defense mechanism in EAC-treated mice. Consistent with our findings, several reports have indicated that thiazole derivatives with potential anticancer activity act by increasing enzymatic antioxidant activity (catalase, GSH) and reducing MDA in hepatic and renal tissues of EAC-treated models.^33,115^ Thiols are also very important antioxidants that protect cells against oxidative stress through their high binding affinity for metals.^116^

Inoculation and invasion of EAC cells are associated with alterations in metabolism, inflammation, fibrosis, and toxicity to organs during tumor development.^117,118^ Our biochemical and histological examinations revealed that the EAC-induced group exhibited massive damage to liver and kidney function and tissue architecture. Markedly, the administration of BTHP (5 mg per kg per day, 14 days) significantly improved liver and kidney functions in the EAC-induced group, as indicated by significant decreases in the levels of serum ALT, AST, creatinine and blood urea, but also significant increases in the serum ALB and total protein levels. Several studies documented the cytoprotective role of numerous anticancer thiazoles on liver and kidney function in EAC-bearing mice, such as CP1 and CP2 (ref. 101) and TAP-07 and TP-07.^119^ In contrast, some reports showed that thiazoles impair liver functions with slight changes in kidney functions.^120^ Taken together, our findings indicate that BTHP exerts cytoprotective effects on the kidney and liver. Furthermore, our results underscore the selectivity of BTHP toward cancer cells, shedding light on an attractive alternative treatment to classical anticancer therapies.^11,121^

TGF-β is an inflammatory mediator that plays a basic role in the progression of different cancer cell types and protects cancer cells against programmed cell death.^122,123^ In addition, it can suppress the expression of cytotoxic T cells, which are important effectors of antitumor immune responses.^124,125^ NF-kβ is a proinflammatory transcription factor that is an apoptosis inhibitor involved in tumorigenesis of cancer cells and resistance to anticancer treatment.^126,127^ Both TGF-β and NF-kβ are key regulatory factors or mediators of IL-6.^128,129^ IL-6 is a protumorigenic cytokine involved in the development, proliferation, and expansion of undifferentiated cancerous cells and promotes resistance to some cytotoxic chemotherapies.^130,131^ It is well known that microenvironmental IL-6 inhibits anticancer immune responses.^132,133^ This evidence may explain the increase in the levels of the investigated proinflammatory cytokines (TGF-β, NF-kβ, and IL-6) in the EAC-treated model. Our study indicated that treatment of the EAC-induced model with BTHP (5 mg per kg per day, 14 days) substantially mitigated the expression levels of TGF-β, NF-κB, and IL6 in liver and kidney tissues compared to those in the EAC-treated model, underscoring its anti-inflammatory potential. Several studies have reported the potential anticancer effects of thiazoles through the downregulation of TGF-β, NF-kβ, and IL-6 signaling.^134–136^ Overall, these findings underscore the therapeutic efficiency of BTHP for the development of promising anticancer and anti-inflammatory candidates.

Our study further demonstrated that the anticancer activity of BTHP in the EAC-induced mouse model is closely associated with its ability to modulate apoptotic signaling pathways. Consistent with previous studies emphasizing the role of apoptotic dysregulation in cancer progression,^137^ our results showed significant alterations in the expression levels of key apoptotic proteins in the EAC-treated group. Specifically, the observed downregulation of p53, Bax, caspase-3, and cytochrome c proteins, coupled with elevated Bcl-2 levels, aligns with the anti-apoptotic shift typically seen in tumor cells. Treatment with BTHP (5 mg per kg per day for 14 days) significantly reversed these alterations, restoring pro-apoptotic signaling and suppressing anti-apoptotic mechanisms. The elevation of p53 levels, a key regulator of apoptosis and tumor suppression,^138^ in the BTHP-treated EAC group suggests its pivotal role in reactivating apoptotic pathways. Furthermore, the concurrent upregulation of Bax, cytochrome c, and caspase-3, alongside the suppression of Bcl-2, underscores the activation of intrinsic apoptotic signaling.^139^ The STRING database analysis further emphasized these findings, highlighting strong protein–protein interactions among the studied targets and their critical role in regulating apoptosis. Compounds containing the 1,3-thiazole scaffold are known to interact with various cellular targets, enhancing apoptosis through the modulation of key regulatory proteins such as p53, Bax, and caspase-3.^140^ Previous studies have shown that 1,3-thiazoles can induce mitochondrial membrane potential loss and cytochrome c release, leading to caspase activation and programmed cell death. Additionally, the suppression of anti-apoptotic proteins like Bcl-2 by 1,3-thiazoles has been associated with their ability to sensitize tumor cells to apoptotic stimuli, thereby overcoming resistance mechanisms.^140,141^ Our study aligns with these established properties of 1,3-thiazoles, further validating BTHP's mechanism of action as a potential apoptosis inducer. Furthermore, the nonsignificant effects on apoptotic markers in the control group treated with BTHP highlight its selectivity and safety profile.

The histopathological examinations revealed substantial damage to the kidney and liver tissues of the EAC-bearing group that could be correlated to the accumulation of hemorrhagic ascitic fluid, tumor metastasis, and invasion of tumor cells.^142–144^ Consistent with our biochemical analysis, our findings revealed a decrease in the serum ALB and total protein levels and an increase in the AST, ALT, urea, and creatinine levels in the EAC-induced group, suggesting liver and kidney dysfunctions. Thus, our histopathological examinations aligned with the biochemical findings of liver and kidney function in the EAC groups. Interestingly, the histopathological assessments revealed significant histological improvements in the renal and liver tissue of the BTHP-treated EAC-induced group. Thiazole derivatives are known to have hepatorenal protective effects, as they improve EAC-induced histopathological changes.^33^ In agreement with the renal and hepatic protective effects of BTHP, an array of thiazole derivatives, including TD1, CP1, and CP2, also ameliorated the changes in liver and kidney tissues in EAC-treated models.^33,145^ This improvement suggested that BTHP played a substantial role in alleviating the hepatorenal damage induced by EAC cells, restoring structural integrity and functional capacity.

## 5. Conclusion

Thiazoles, including our novel 1,3-thiazole analog BTHP, hold significant promise as anticancer agents due to their structural diversity and potential for rational drug design. Building on our previous findings demonstrating the potent antiproliferative activity of BTHP against breast cancer cells *via* targeting vascular endothelial growth factor receptor-2, our current study investigated its therapeutic potential in an EAC-induced murine model. The results revealed that BTHP significantly inhibited EAC progression by reducing tumor volume and viable cell count while markedly extending lifespan compared to untreated EAC-bearing mice. Mechanistically, BTHP restored oxidative balance by enhancing antioxidant enzyme levels (GSH, SOD, and catalase) and reducing lipid peroxidation (MDA). BTHP further preserved hepatorenal function by attenuating EAC-induced liver and kidney damage, as demonstrated by improved serum biomarkers (ALT, AST, urea, creatinine, albumin, and total protein) and histological integrity. The compound also exhibited potent anti-inflammatory effects, significantly downregulating proinflammatory markers (TGF-β, NFκB, and IL6) in liver and kidney tissues. Notably, BTHP corrected the apoptotic dysregulation induced by EAC, upregulating pro-apoptotic markers (p53, Bax, cytochrome c, and caspase-3) and downregulating the anti-apoptotic protein Bcl-2. This dual modulation of apoptosis and inflammation underscores BTHP's ability to combat cancer through multiple synergistic pathways. Molecular modeling studies further supported these findings, revealing strong binding interactions between BTHP and key protein targets associated with its anticancer mechanisms. Collectively, these results highlight BTHP as a promising therapeutic agent with multifaceted mechanisms of action, including antioxidant, anti-inflammatory, pro-apoptotic, and organ-protective effects. These findings pave the way for further investigations into BTHP's potential as a targeted anticancer therapy. To further elucidate its therapeutic potential, future research should prioritize a detailed investigation into the specific molecular mechanisms underlying these effects. Additionally, expanding the evaluation of BTHP's efficacy across a wider range of cancer models is imperative to comprehensively validate its therapeutic utility. In this context, the application of multiomics approaches, such as metabolomics and proteomics, could provide critical insights into the molecular targets modulated by BTHP. These targets can subsequently be verified through rigorous molecular assessments, thereby advancing our understanding of BTHP's precise therapeutic mechanisms. Ultimately, BTHP has the potential to contribute significantly to ongoing efforts to develop effective and safer cancer treatments.

## Ethical statement

The animal study protocol was approved by the Animal Ethical Committee of the Faculty of Pharmacy, Suez Canal University, Egypt (Approval No. 202111MA1).

## Author contributions

Conceptualization, M. E. B., D. M. A., M. A., D. I. M., E. R. S., M. H. H., E. A.-O., I. A. A. I., S. M., and E. M. S.; methodology, M. E. B., D. M. A., M. A., Y. I. M. E., D. I. M., E. R. S., M. H. H., E. A.-O., I. A. A. I., G. A. B., S. M., and E. M. S.; software, Y. I. M. E., E. R. S., M. H. H., E. A.-O., I. A. A. I., G. A. B., S. M., and E. M. S.; validation, M. E. B., D. M. A., Y. I. M. E., D. I. M., M. H. H., E. A.-O., G. A. B., S. M., and E. M. S.; formal analysis, M. E. B., D. M. A., M. A., Y. I. M. E., D. I. M., E. R. S., E. A.-O., I. A. A. I., S. M., and E. M. S.; investigation, M. E. B., D. M. A., M. A., D. I. M., E. R. S., I. A. A. I., G. A. B., and E. M. S.; resources, M. E. B., D. M. A., M. A., Y. I. M. E., D. I. M., E. R. S., M. H. H., E. A.-O., I. A. A. I., G. A. B., S. M., and E. M. S.; data curation, M. E. B., D. M. A., M. A., Y. I. M. E., D. I. M., M. H. H., G. A. B., S. M., and E. M. S.; writing—original draft preparation M. E. B., D. M. A., M. A., Y. I. M. E., D. I. M., E. R. S., M. H. H., E. A.-O., I. A. A. I., G. A. B., S. M., and E. M. S.; writing—review and editing, M. E. B., D. M. A., M. A., Y. I. M. E., D. I. M., E. R. S., M. H. H., E. A.-O., I. A. A. I., G. A. B., S. M., and E. M. S.; visualization, M. E. B., D. M. A., M. A., Y. I. M. E., D. I. M., E. R. S., M. H. H., E. A.-O., I. A. A. I., G. A. B., S. M., and E. M. S.; supervision, M. E. B., D. M. A., D. I. M., M. H. H., E. A.-O., I. A. A. I., S. M., and E. M. S.; project administration, M. E. B., D. M. A., D. I. M., M. H. H., S. M., and E. M. S.; funding acquisition, M. E. B., D. M. A., M. A., D. I. M., E. A.-O., I. A. A. I., G. A. B., and E. M. S. All authors have read and agreed to the published version of the manuscript.

## Conflicts of interest

The authors affirm that they are not aware of any personal or financial conflicts that would have seemed to affect the findings of this study's research.

## Supplementary Material

RA-015-D5RA01014D-s001

RA-015-D5RA01014D-s002

RA-015-D5RA01014D-s003

RA-015-D5RA01014D-s004

RA-015-D5RA01014D-s005

RA-015-D5RA01014D-s006

RA-015-D5RA01014D-s007

RA-015-D5RA01014D-s008

## Data Availability

Data supporting the results reported in this manuscript are included in this article and as part of the ESI.† The raw data supporting the conclusions of this article will be made available by the authors without any undue reservation.
