# Supplementary figures and images for: Exploration of the anticancer efficacy of a novel 1,3-thiazole analog in an ehrlich ascites carcinoma model: in vivo and in silico insights into hepatorenal protective potentials via the modulation of apoptosis, oxidative stress and inflammation
Source: RSC Adv. 2025 Jun 13;15(25):20143–67. doi: 10.1039/d5ra01014d (PMC12163905; doi:10.1039/d5ra01014d)

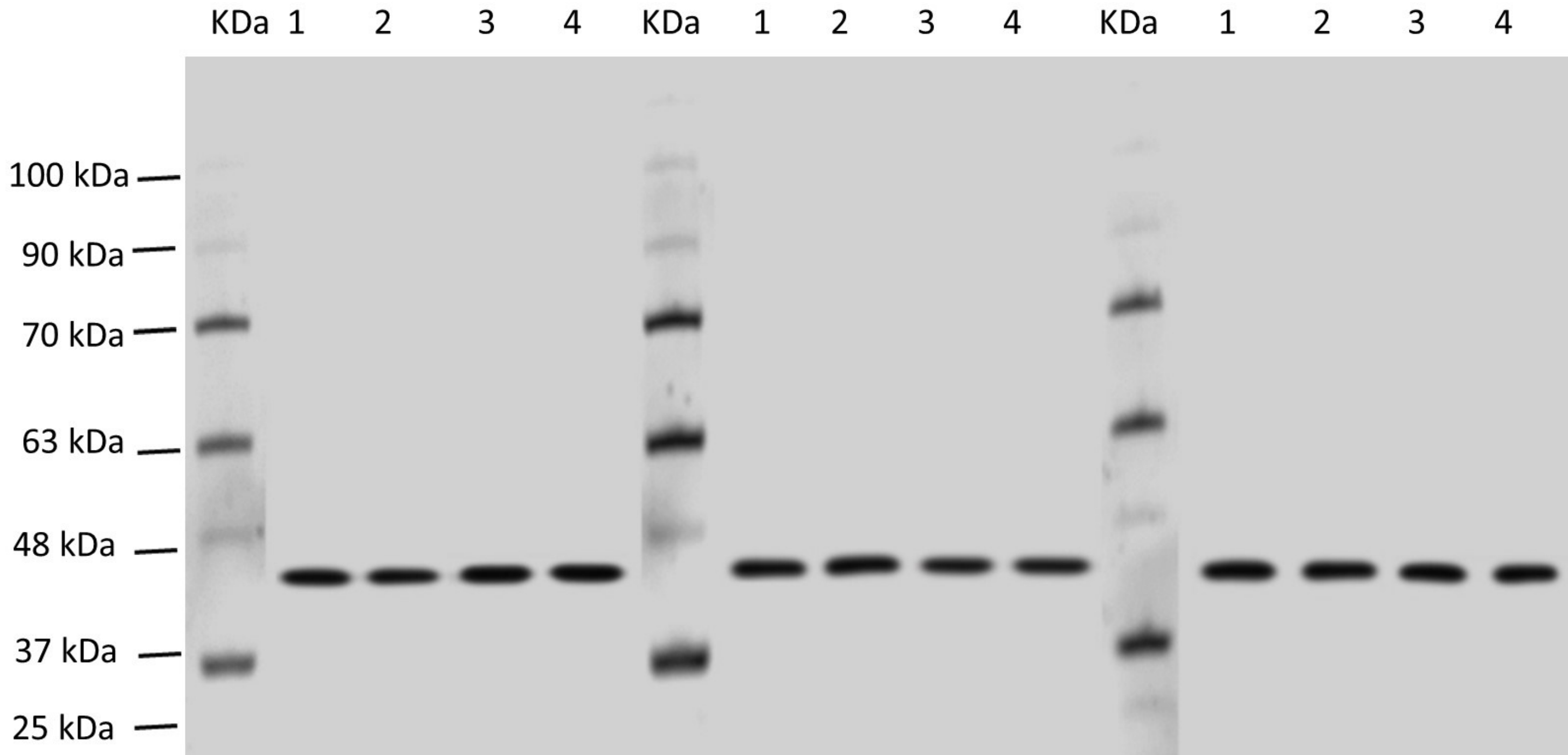

Supplement: RA-015-D5RA01014D-s001 [file RA-015-D5RA01014D-s001.pdf]

KDa 1 2 3 4 KDa 1 2 3 4 KDa 1 2 3 4

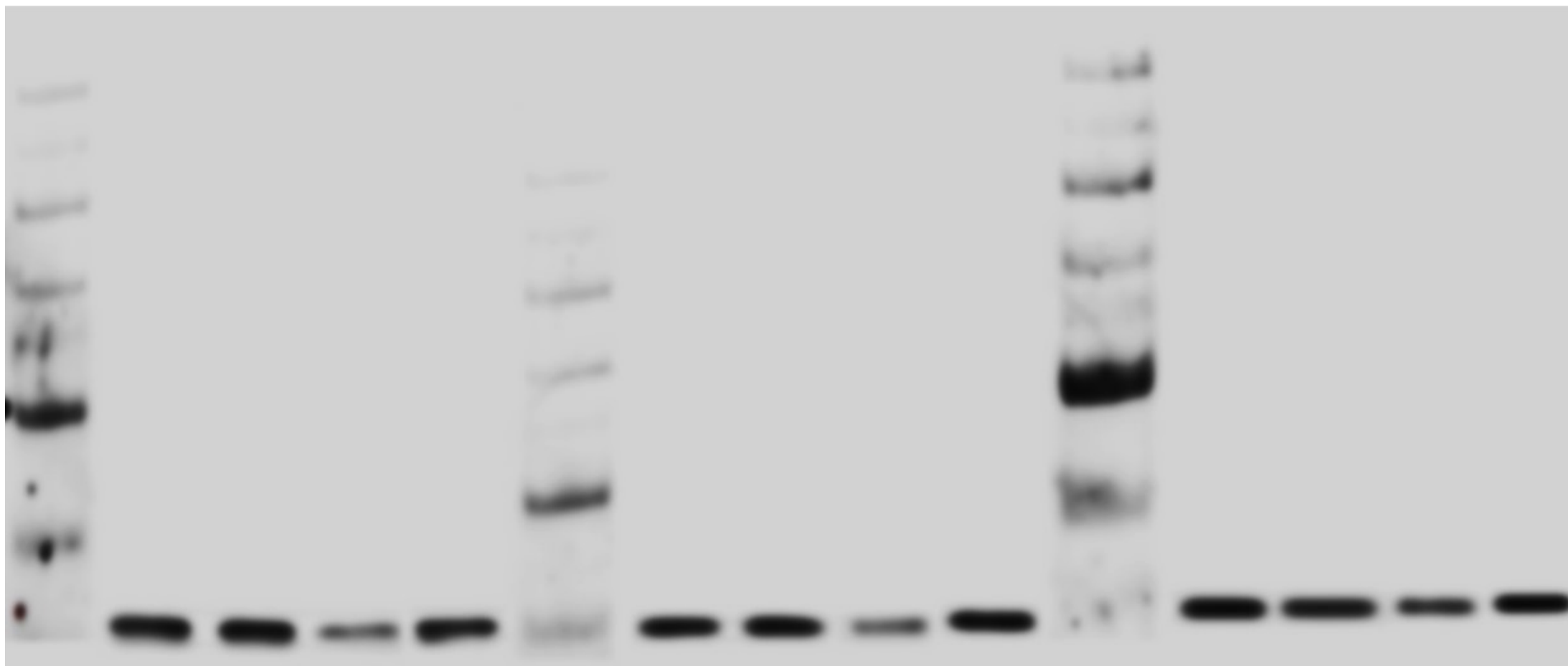

Supplement: RA-015-D5RA01014D-s002 [file RA-015-D5RA01014D-s002.pdf]

KDa 1 2 3 4 KDa 1 2 3 4 KDa 1 2 3 4

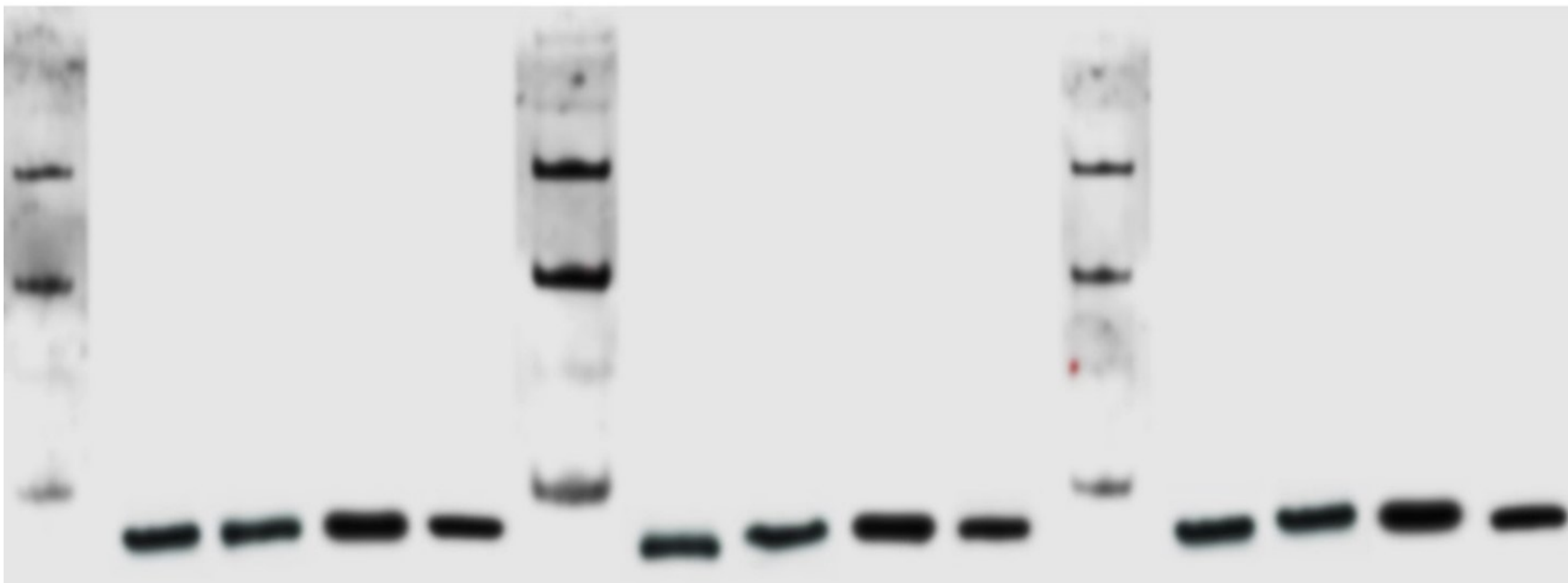

Supplement: RA-015-D5RA01014D-s003 [file RA-015-D5RA01014D-s003.pdf]

KDa 1 2 3 4 KDa 1 2 3 4 KDa 1 2 3 4

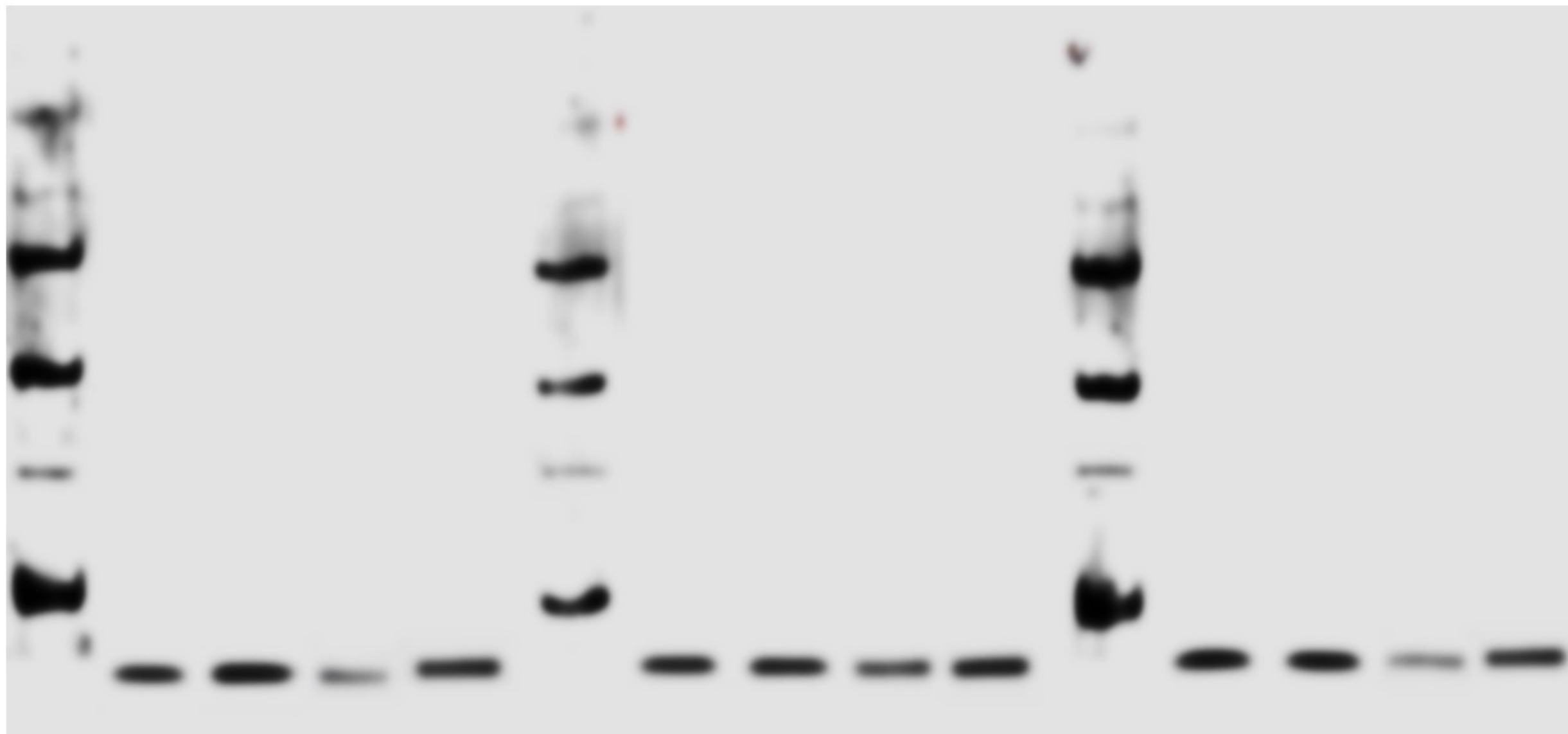

Supplement: RA-015-D5RA01014D-s004 [file RA-015-D5RA01014D-s004.pdf]

KDa 1 2 3 4 KDa 1 2 3 4 KDa 1 2 3 4

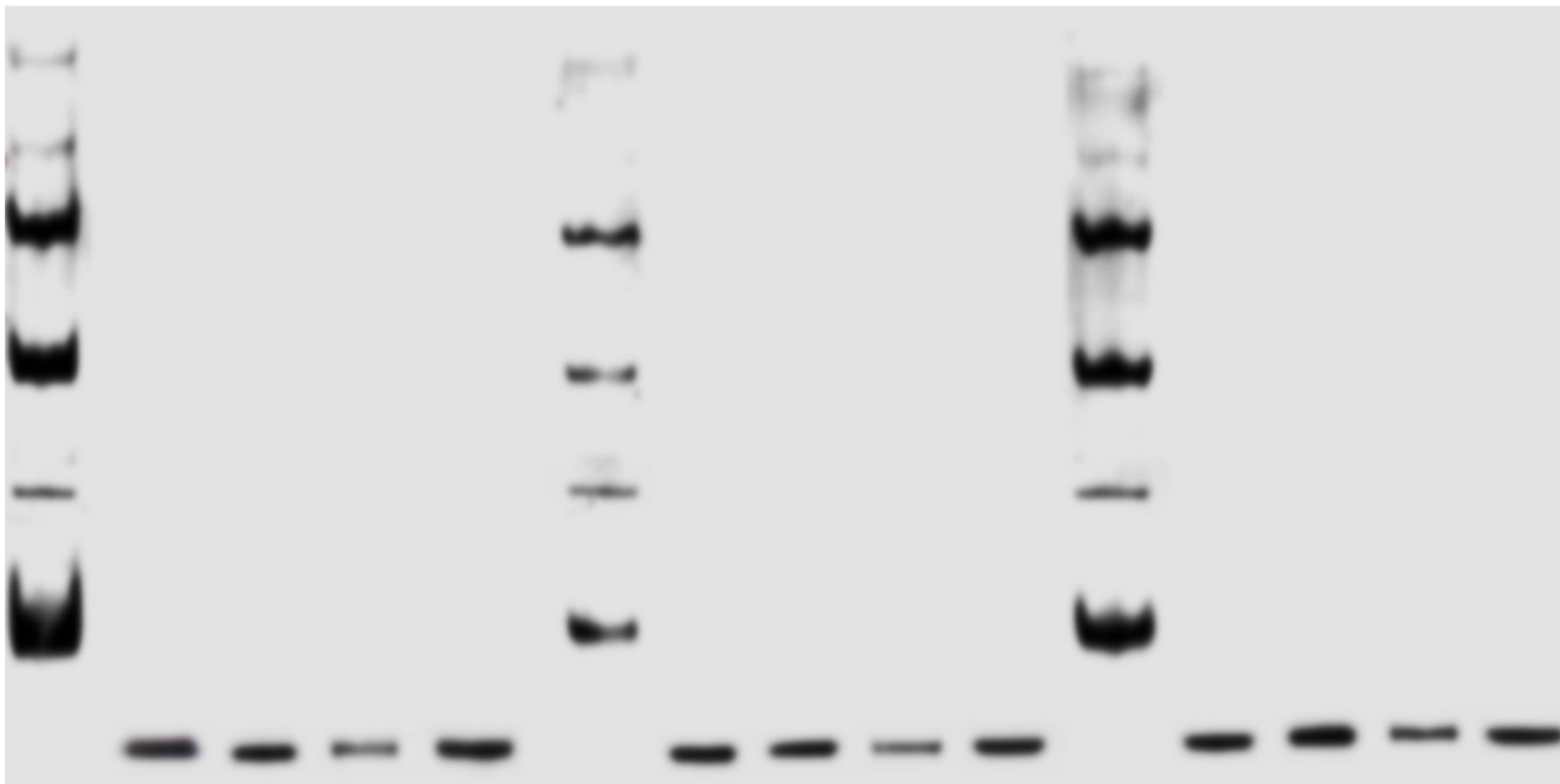

Supplement: RA-015-D5RA01014D-s005 [file RA-015-D5RA01014D-s005.pdf]

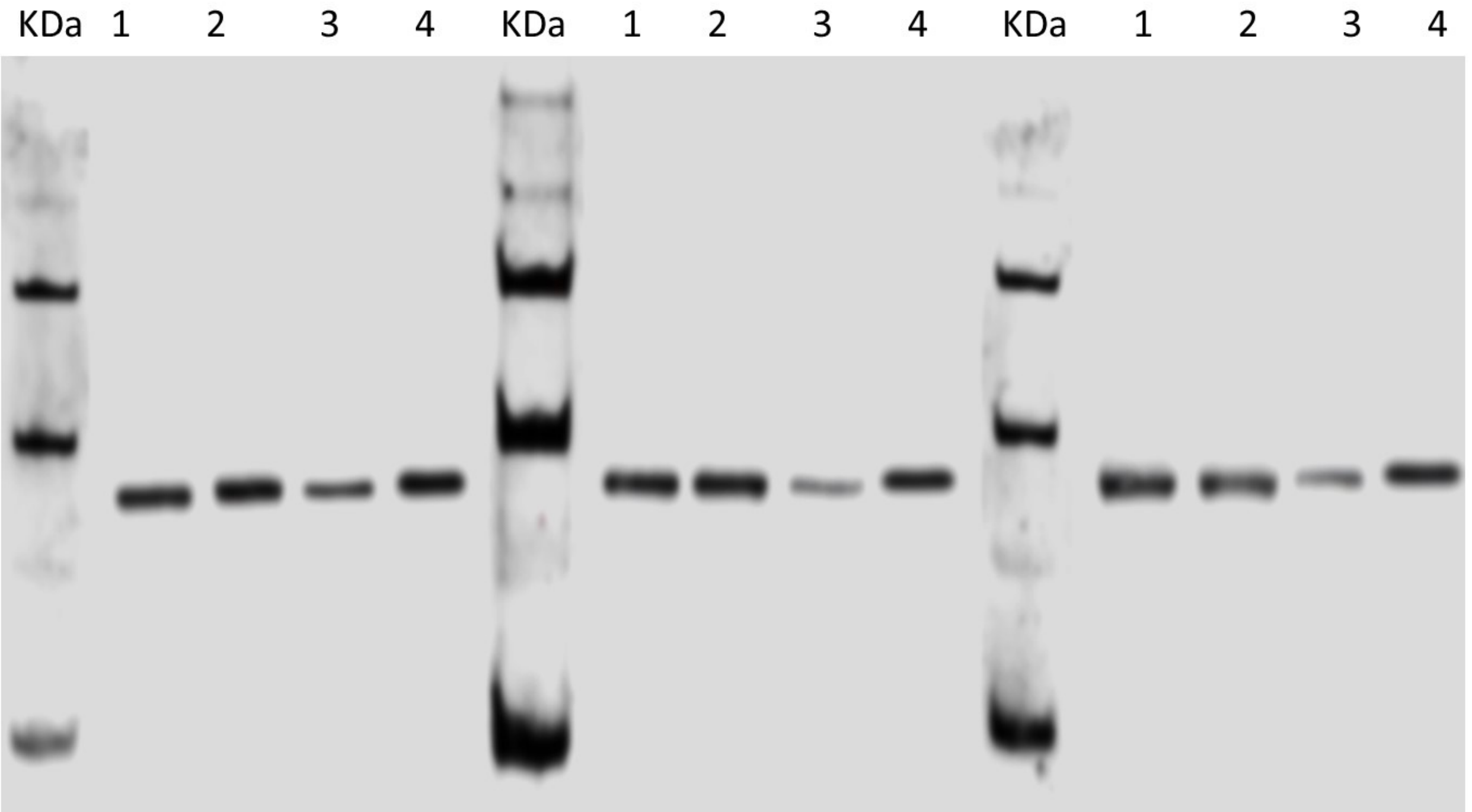

Supplement: RA-015-D5RA01014D-s007 [file RA-015-D5RA01014D-s007.pdf]
