# Supplementary material for: Exploration of the anticancer efficacy of a novel 1,3-thiazole analog in an ehrlich ascites carcinoma model: in vivo and in silico insights into hepatorenal protective potentials via the modulation of apoptosis, oxidative stress and inflammation
Source: RSC Adv. 2025 Jun 13;15(25):20143–67. doi: 10.1039/d5ra01014d (PMC12163905; doi:10.1039/d5ra01014d)
Supplement: RA-015-D5RA01014D-s008 [file RA-015-D5RA01014D-s008.pdf]

Supplementary Information (SI) for RSC Advances.
This journal is © The Royal Society of Chemistry 2025

Supporting Information

Exploration of the Anticancer Efficacy of a Novel 1,3-Thiazole Analog in an Ehrlich Ascites Carcinoma Model: Insights into Hepatorenal Protective Potentials via the Modulation of Apoptosis, Oxidative Stress and Inflammation

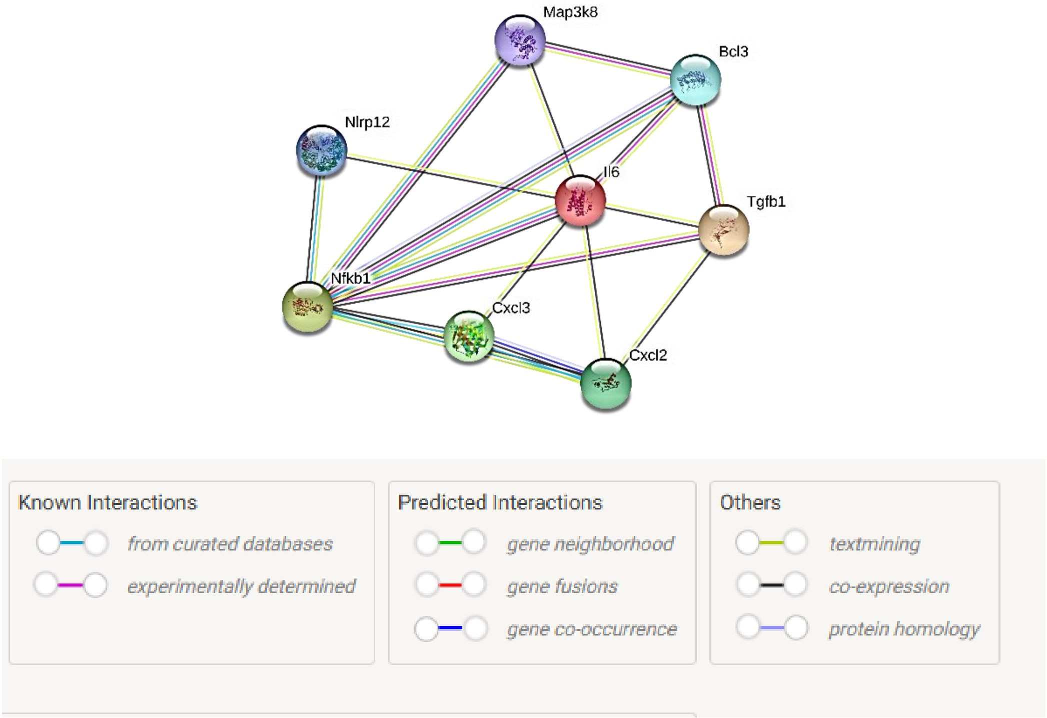

Figure S1: A typical association network in STRING for computational protein interaction analysis of studied targets. The colored nodes denote the proteins, while the edges signify the protein-protein association as shown in the legend section. The interacting nodes with the central hub IL6 represented the predicted functional protein partners that include "NF-kβ 1" Nuclear factor NF-kappa-B p105 subunit, 'TGFB Transforming growth factor beta-1 proprotein, "Cxcl3" C-X-C motif chemokine 3, "Cxcl2" C-X-C motif chemokine 2, "Bcl3" B-cell lymphoma 3 protein homolog, 'Nlrp12' NACHT, LRR and PYD, domains-containing protein 12, 'Map3k8', Mitogen-activated protein kinase 8 (**Table S2**).

https://version-12-0.string-db.org/cgi/network?networkId=bA0MMxvoAPWZ.

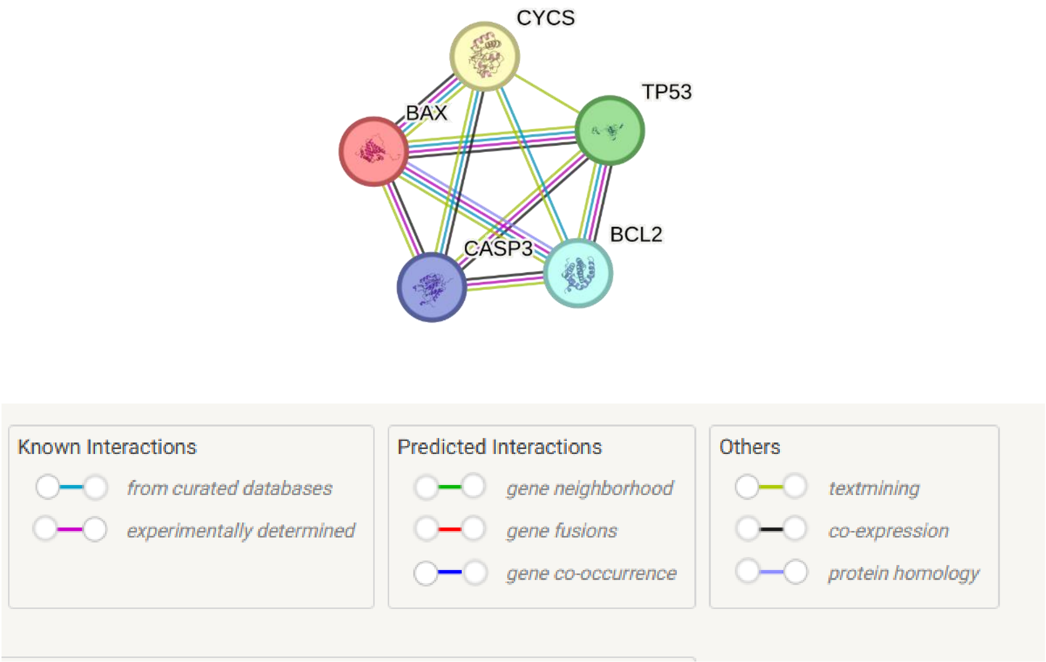

Figure S2: A typical association network in STRING for computational protein interaction analysis of studied apoptotic targets. The colored nodes denote the proteins, while the edges signify the protein-protein association as shown in the legend section. The interacting nodes with the central hub p53 'TP53'represented the predicted functional protein partners that include Apoptosis regulator BAX "BAX", 'Cytochrome c, "CYCS", Caspase-3 subunit p12 "CASP3", and apoptosis regulator Bcl-2, 'BCL2' (**Table S3**).

https://version-12-0.string-db.org/cgi/network?taskId=bL11f6y0nNPa&sessionId=bFOZWno1LXYK

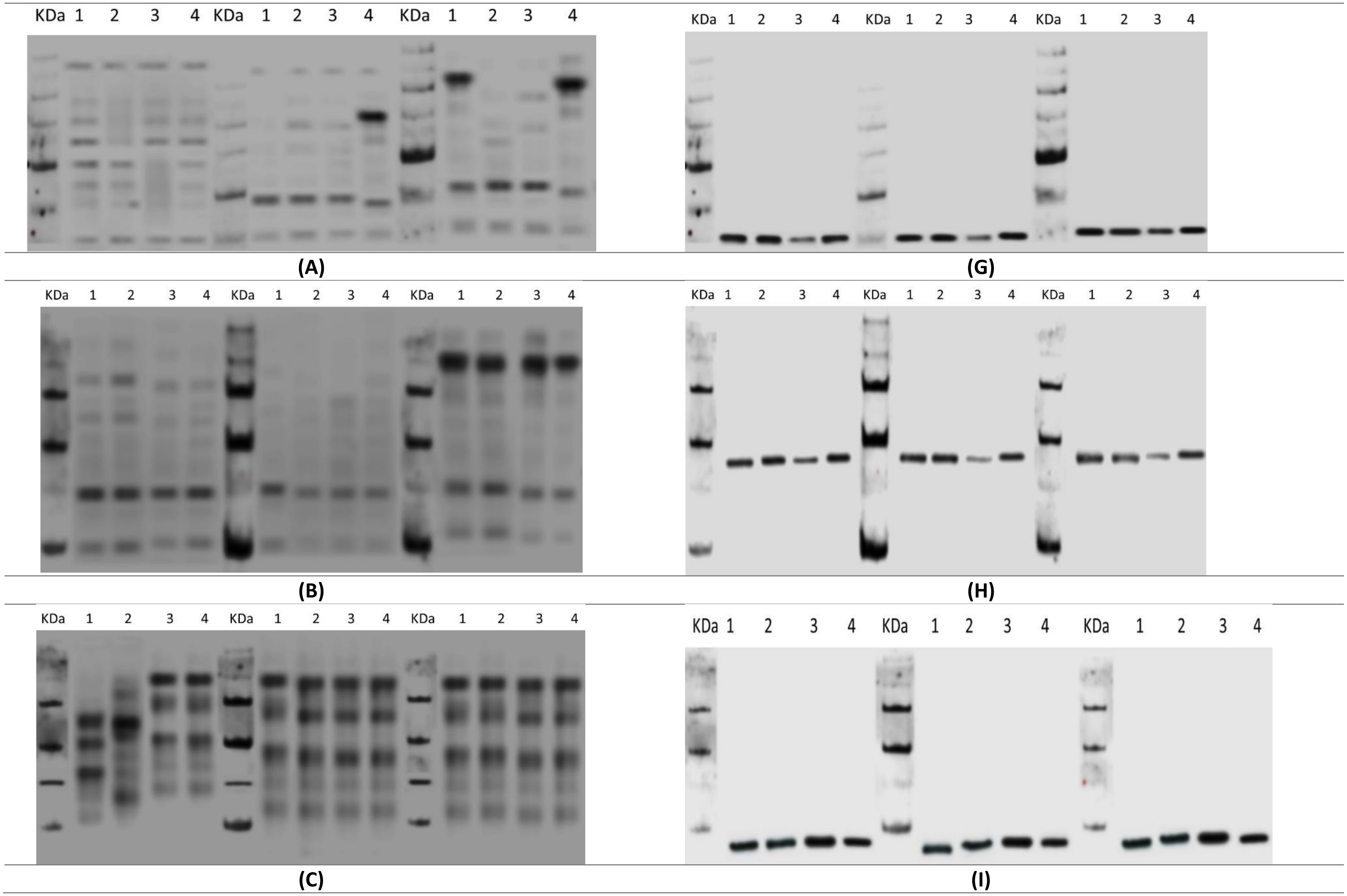
KDa 1 2 3 4 KDa 1 2 3 4 KDa 1 2 3 4
(A)
KDa 1 2 3 4 KDa 1 2 3 4 KDa 1 2 3 4
(G)
KDa 1 2 3 4 KDa 1 2 3 4 KDa 1 2 3 4
(B)
KDa 1 2 3 4 KDa 1 2 3 4 KDa 1 2 3 4
(H)
KDa 1 2 3 4 KDa 1 2 3 4 KDa 1 2 3 4
(C)
KDa 1 2 3 4 KDa 1 2 3 4 KDa 1 2 3 4
(I)

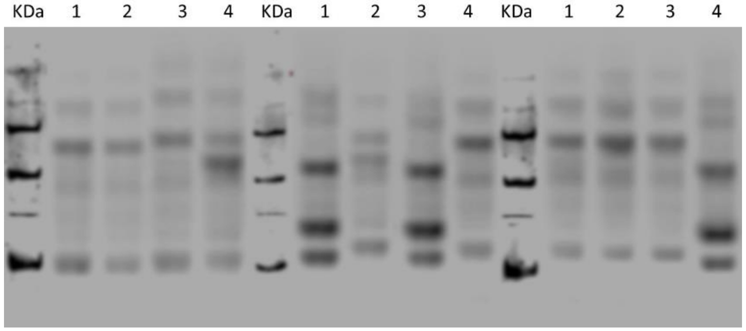
KDa 1 2 3 4 KDa 1 2 3 4 KDa 1 2 3 4

(D)

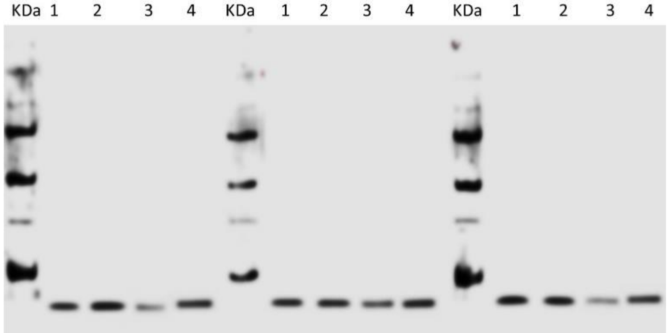
KDa 1 2 3 4 KDa 1 2 3 4 KDa 1 2 3 4

(J)

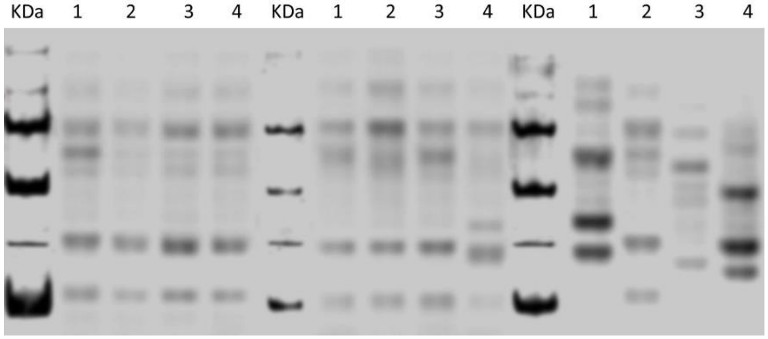
KDa 1 2 3 4 KDa 1 2 3 4 KDa 1 2 3 4

(E)

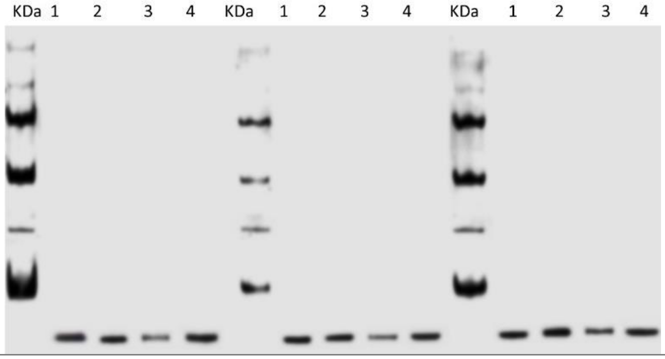
KDa 1 2 3 4 KDa 1 2 3 4 KDa 1 2 3 4

(K)

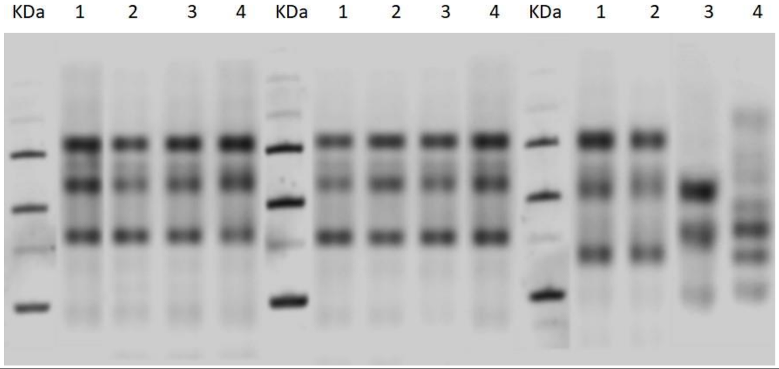
KDa 1 2 3 4 KDa 1 2 3 4 KDa 1 2 3 4

(F)

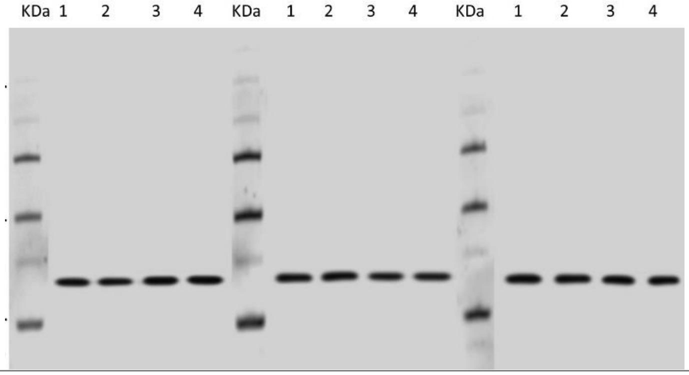
KDa 1 2 3 4 KDa 1 2 3 4 KDa 1 2 3 4

(L)

Figure S3: Effect of BTHP treatment (5 mg/kg) on the protein expression of the apoptotic markers Bax (**A, G**), p53 (**B, H**), Bcl-2 (**C, I**), Caspase 3 (**D, J**), and cytochrome C (**E, K**) in EAC-induced model. (**F, L**) represents the reference β-actin. (**A-F**) represents the western blots and (**G-L**) represents chemiluminescent images for the examined markers.

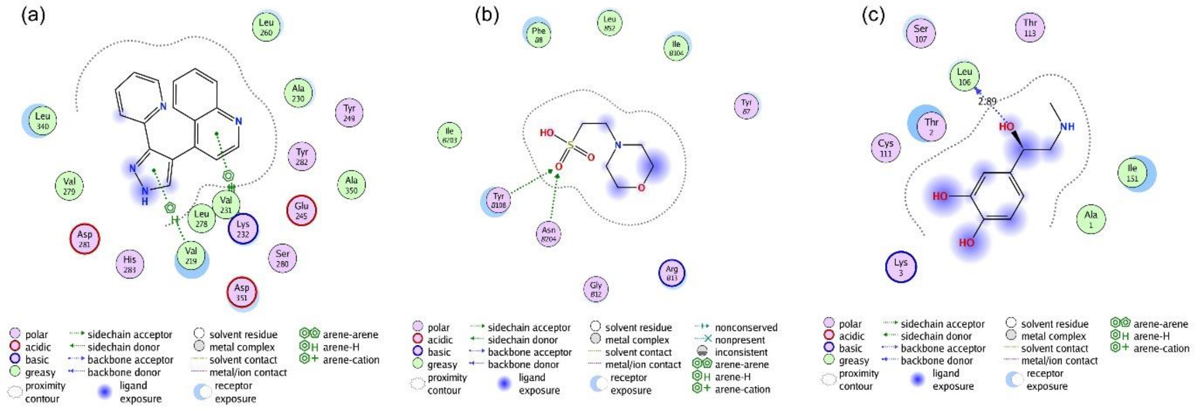

Figure S4. The 2D binding interactions for native co-crystallized ligands against targets (a) TGF-β; (b) Glutathione transferase P1-1 (GSTP1-1); and (c) superoxide dismutase-1 (SOD1), respectively.

Table S1: The list of primer pairs of proinflammatory genes utilized in qRT-PCR analysis

Gene Name	Forward primer	Reverse primers
**MUS IL-6,**	F5′-GAGGATACCACTCCCAACAGACC-3′	R5′-AAGTGCATCATCGTTGTTCATACA -3′
**MUS TGF-β**	F5’- TGATACGCCTGAGTGGCTGTCT -3’	R 5- CACAAGAGCAGTGAGCGCTGAA -3`
**MUS NFKB**	F 5’- GCTGCCAAAGAAGGACACGACA -3’	R 5'- GGCAGGCTATTGCTCATCACAG -3'
**MUS GAPDH**	F 5’- ACCCAGAAGACTGTGGATGG -3’	R5'- ACACATTGGGGGTAGGAACA -3'

Table S2: Network Interaction Scores of Inflammatory Pathway Genes in Protein-Protein Interactions

node1	node2	node1 accession	node2 accession	node1 annotation	node2 annotation	score
**Tgfb1**	Nfkb1	ENSMUSP00000002678	ENSMUSP00000029812	*Transforming growth factor beta-1 proprotein; Transforming growth factor beta-1 proprotein: Precursor of the Latency-associated peptide (LAP) and Transforming growth factor beta-1 (TGF-beta-1) chains, which constitute the regulatory and active subunit of TGF-beta-1, respectively. Transforming growth factor beta-1: Multifunctional protein that regulates the growth and differentiation of various ce types and is involved in various processes, such as normal development, immune function, microglia function and responses to neurodegeneration. Activation into mature form follows different [...]*	*Nuclear factor NF-kappa-B p105 subunit; NF-kappa-B is a pleiotropic transcription factor present in almost all cell types and is the endpoint of a series of signal transduction events that are initiated by a vast array of stimuli related to many biological processes such as inflammation, immunity, differentiation, cell growth, tumorigenesis and apoptosis. NF-kappa-B is a homo- or heterodimeric complex formed by the Rel-like domain- containing proteins RELA/p65, RELB, NFKB1/p105, NFKB1/p50, REL and NFKB2/p52 and the heterodimeric p65-p50 complex appears to be most abundant one. The dime [...]*	0.534
**Tgfb1**	Il6	ENSMUSP00000002678	ENSMUSP00000026845	*Transforming growth factor beta-1 proprotein: Precursor of the Latency-associated peptide (LAP) and Transforming growth factor beta-1 (TGF-beta-1) chains, which constitute the regulatory and active subunit of TGF-beta-1, respectively. Transforming growth factor beta-1: Multifunctional protein that regulates the growth and differentiation of various cell types and is involved in various processes, such as normal development, immune function, microglia function and responses to neurodegeneration.*	*Interleukin-6; Cytokine with a wide variety of biological functions. It is a potent inducer of the acute phase response. Plays an essential role in the final differentiation of B-cells into Ig-secreting cells Involved in lymphocyte and monocyte differentiation. Acts on B-cells, T-cells, hepatocytes, hematopoietic progenitor cells and cells of the CNS. Required for the generation of T(H)17 cells. Also acts as a myokine. It is discharged into the bloodstream after muscle contraction and acts to increase the breakdown of fats and to improve insulin resistance*	0.892
**Tgfb1**	Cxcl2	ENSMUSP00000002678	ENSMUSP00000074885	*Transforming growth factor beta-1 proprotein: Precursor of the Latency-associated peptide (LAP) and Transforming growth factor beta-1 (TGF-beta-1) chains, which constitute the regulatory and active subunit of TGF-beta-1, respectively. Transforming growth factor beta-1: Multifunctional protein that regulates the growth and differentiation of various cell types and is involved in various processes, such as normal development, immune function, microglia function and responses to neurodegeneration.*	*C-X-C motif chemokine 2; Chemotactic for human polymorphonuclear leukocytes but does not induce chemokinesis or an oxidative burst.*	0.449
**Tgfb1**	BCL3	ENSMUSP00000002678	ENSMUSP00000113851	*Transforming growth factor beta-1 proprotein: Precursor of the Latency-associated peptide (LAP) and Transforming growth factor beta-1 (TGF-beta-1) chains, which constitute the regulatory and active subunit of TGF-beta-1, respectively. Transforming growth factor beta-1: Multifunctional protein that regulates the growth and differentiation of various cell types and is involved in various processes, such as normal development, immune function, microglia function and responses to neurodegeneration.*	*B-cell lymphoma 3 protein homolog; Contributes to the regulation of transcriptional activation of NF-kappa-B target genes. In the cytoplasm, inhibits the nuclear translocation of the NF-kappa-B p50 subunit (By similarity). In the nucleus, acts as transcriptional activator that promotes transcription of NF-kappa-B target genes. Contributes to the regulation of cell proliferation.*	0.553
**Nlrp12**	Nfkb1	ENSMUSP00000104293	ENSMUSP00000029812	*NACHT, LRR and PYD domains-containing protein 12; Plays an essential role as an potent mitigator of*	*Nuclear factor NF-kappa-B p105 subunit; NF-kappa-B is a pleiotropic transcription factor present in almost all cell types*	0.927

				inflammation. Primarily expressed in dendritic cells and macrophages, inhibits both canonical and non-canonical NF-kappa-B and ERK activation pathways. Functions as a negative regulator of NOD2 by targeting it to degradation via the proteasome pathway. In turn, promotes bacterial tolerance. Inhibits also the DDX58-mediated immune signaling against RNA viruses by reducing the E3 ubiquitin ligase TRIM25-mediated 'Lys-63'-linked DDX58 activation but enhancing the E3 ubiquitin ligase RNF125 [...]	*and is the endpoint of a series of signal transduction events that are initiated by a vast array of stimuli related to many biological processes such as inflammation, immunity, differentiation, cell growth, tumorigenesis and apoptosis. NF-kappa-B is a homo- or heterodimeric complex formed by the Rel-like domain- containing proteins RELA/p65, RELB, NFKB1/p105, NFKB1/p50, REL and NFKB2/p52 and the heterodimeric p65-p50 complex appears to be most abundant one.*	
**Nlrp12**	Il6	ENSMUSP00000104293	ENSMUSP00000026845	*NACHT, LRR and PYD domains-containing protein 12; Plays an essential role as an potent mitigator of inflammation. Primarily expressed in dendritic cells and macrophages, inhibits both canonical and non- canonical NF-kappa-B and ERK activation pathways. Functions as a negative regulator of NOD2 by targeting it to degradation via the proteasome pathway. In turn, promotes bacterial tolerance. Inhibits also the DDX58-mediated immune signaling against RNA viruses by reducing the E3 ubiquitin ligase TRIM25-mediated 'Lys-63'-linked DDX58 activation but enhancing the E3 ubiquitin ligase RNF125 [...]*	*Interleukin-6; Cytokine with a wide variety of biological functions. It is a potent inducer of the acute phase response. Plays an essential role in the final differentiation of B-cells into Ig-secreting cells Involved in lymphocyte and monocyte differentiation. Acts on B-cells, T-cells, hepatocytes, hematopoietic progenitor cells and cells of the CNS. Required for the generation of T(H)17 cells. Also acts as a myokine. It is discharged into the bloodstream after muscle contraction and acts to increase the breakdown of fats and to improve insulin resistance*	0.407
**Nfkb1**	Tgfb1	ENSMUSP00000029812	ENSMUSP00000002678	*Nuclear factor NF-kappa-B p105 subunit; NF-kappa-B is a pleiotropic transcription factor present in almost all cell types and is the endpoint of a series of signal transduction events that are initiated by a vast array of stimuli related to many biological processes such as inflammation, immunity, differentiation, cell growth, tumorigenesis and apoptosis. NF-kappa-B is a homo- or heterodimeric complex formed by the Rel-like domain- containing proteins RELA/p65, RELB, NFKB1/p105, NFKB1/p50, REL and NFKB2/p52 and the heterodimeric p65-p50 complex appears to be most abundant one. The dime [...]*	*Transforming growth factor beta-1 proprotein; Transforming growth factor beta-1 proprotein: Precursor of the Latency-associated peptide (LAP) and Transforming growth factor beta-1 (TGF-beta-1) chains, which constitute the regulatory and active subunit of TGF-beta-1, respectively. Transforming growth factor beta-1: Multifunctional protein that regulates the growth and differentiation of various cell types and is involved in various processes, such as normal development, immune function, microglia function and responses to neurodegeneration. Activation into mature form follows different [...]*	0.534
**Nfkb1**	Nlrp12	ENSMUSP00000029812	ENSMUSP00000104293	*Nuclear factor NF-kappa-B p105 subunit; NF-kappa-B is a pleiotropic transcription factor present in almost all cell types and is the endpoint of a series of signal transduction events that are initiated by a vast array of stimuli related to many biological processes such as inflammation, immunity, differentiation, cell growth, tumorigenesis and apoptosis. NF-kappa-B is a homo- or heterodimeric complex formed by the Rel-like domain- containing proteins RELA/p65, RELB, NFKB1/p105, NFKB1/p50, REL and NFKB2/p52 and the heterodimeric p65-p50*	*NACHT, LRR and PYD domains-containing protein 12; Plays an essential role as an potent mitigator of inflammation. Primarily expressed in dendritic cells and macrophages, inhibits both canonical and non- canonical NF-kappa-B and ERK activation pathways. Functions as a negative regulator of NOD2 by targeting it to degradation via the proteasome pathway. In turn, promotes bacterial tolerance. Inhibits also the DDX58-mediated immune signaling against RNA viruses by reducing the E3 ubiquitin ligase TRIM25-mediated 'Lys-63'-linked DDX58 activation but enhancing the E3 ubiquitin ligase RNF125 [...]*	0.927

				complex appears to be most abundant one. The dime [...]		
**Nfkb1**	Map3k8	ENSMUSP00000029812	ENSMUSP00000025078	*Nuclear factor NF-kappa-B p105 subunit; NF-kappa-B is a pleiotropic transcription factor present in almost all cell types and is the endpoint of a series of signal transduction events that are initiated by a vast array of stimuli related to many biological processes such as inflammation, immunity, differentiation, cell growth, tumorigenesis and apoptosis. NF-kappa-B is a homo- or heterodimeric complex formed by the Rel-like domain- containing proteins RELA/p65, RELB, NFKB1/p105, NFKB1/p50, REL and NFKB2/p52 and the heterodimeric p65-p50 complex appears to be most abundant one. The dime [...]*	*Mitogen-activated protein kinase kinase8; Required for lipopolysaccharide (LPS)-induced, TLR4-mediated activation of the MAPK/ERK pathway in macrophages, thus being critical for production of the proinflammatory cytokine TNF-alpha (TNF) during immune responses. Involved in the regulation of T-helper cell differentiation and IFNG expression in T-cells. Involved in mediating host resistance to bacterial infection through negative regulation of type I interferon (IFN) production. Transduces CD40 and TNFRSF1A signals that activate ERK in B-cells and macrophages, and thus may play a [...]*	0.993
**Nfkb1**	Il6	ENSMUSP00000029812	ENSMUSP00000026845	*Nuclear factor NF-kappa-B p105 subunit; NF-kappa-B is a pleiotropic transcription factor present in almost all cell types and is the endpoint of a series of signal transduction events that are initiated by a vast array of stimuli related to many biological processes such as inflammation, immunity, differentiation, cell growth, tumorigenesis and apoptosis. NF-kappa-B is a homo- or heterodimeric complex formed by the Rel-like domain- containing proteins RELA/p65, RELB, NFKB1/p105, NFKB1/p50, REL and NFKB2/p52 and the heterodimeric p65-p50 complex appears to be most abundant one. The dime [...].*	*Interleukin-6; Cytokine with a wide variety of biological functions. It is a potent inducer of the acute phase response. Plays an essential role in the final differentiation of B-cells into Ig-secreting cells Involved in lymphocyte and monocyte differentiation. Acts on B-cells, T-cells, hepatocytes, hematopoietic progenitor cells and cells of the CNS. Required for the generation of T(H)17 cells. Also acts as a myokine. It is discharged into the bloodstream after muscle contraction and acts to increase the breakdown of fats and to improve insulin resistance*	0.985
**Nfkb1**	Cxcl3	ENSMUSP00000029812	ENSMUSP00000031326	*Nuclear factor NF-kappa-B p105 subunit; NF-kappa-B is a pleiotropic transcription factor present in almost all cell types and is the endpoint of a series of signal transduction events that are initiated by a vast array of stimuli related to many biological processes such as inflammation, immunity, differentiation, cell growth, tumorigenesis and apoptosis. NF-kappa-B is a homo- or heterodimeric complex formed by the Rel-like domain- containing proteins RELA/p65, RELB, NFKB1/p105, NFKB1/p50, REL and NFKB2/p52 and the heterodimeric p65-p50 complex appears to be most abundant one. The dime [...].*	*C-X-C motif chemokine 3; Ligand for CXCR2. Has chemotactic activity for neutrophils. May play a role in inflammation and exert its effects on endothelial cells in an autocrine fashion.*	0.917
**Nfkb1**	Cxcl2	ENSMUSP00000029812	ENSP00000269305	*Nuclear factor NF-kappa-B p105 subunit; NF-kappa-B is a pleiotropic transcription factor present in almost all cell types and is the endpoint of a series of signal transduction events that are initiated by a vast array of stimuli related to many biological processes such as inflammation, immunity, differentiation, cell growth, tumorigenesis and apoptosis. NF-kappa-B is a*	*C-X-C motif chemokine 2; Chemotactic for human polymorphonuclear leukocytes but does not induce chemokinesis or an oxidative burst.*	0.938

				homo- or heterodimeric complex formed by the Rel-like domain- containing proteins RELA/p65, RELB, NFKB1/p105, NFKB1/p50, REL and NFKB2/p52 and the heterodimeric p65-p50 complex appears to be most abundant one. The dime [...].		
**Nkfb**	Bcl3	ENSMUSP00000029812	ENSMUSP00000113851	*Nuclear factor NF-kappa-B p105 subunit; NF-kappa-B is a pleiotropic transcription factor present in almost all cell types and is the endpoint of a series of signal transduction events that are initiated by a vast array of stimuli related to many biological processes such as inflammation, immunity, differentiation, cell growth, tumorigenesis and apoptosis. NF-kappa-B is a homo- or heterodimeric complex formed by the Rel-like domain- containing proteins RELA/p65, RELB, NFKB1/p105, NFKB1/p50, REL and NFKB2/p52 and the heterodimeric p65-p50 complex appears to be most abundant one. The dime [...].*	*B-cell lymphoma 3 protein homolog; Contributes to the regulation of transcriptional activation of NF-kappa-B target genes. In the cytoplasm, inhibits the nuclear translocation of the NF-kappa-B p50 subunit (By similarity). In the nucleus, acts as transcriptional activator that promotes transcription of NF-kappa-B target genes. Contributes to the regulation of cell proliferation.*	0.999
**Map3k8**	Nkfb1	ENSMUSP00000025078	ENSMUSP00000029812	*Mitogen-activated protein kinase kinase kinase 8; Required for lipopolysaccharide (LPS)-induced, TLR4-mediated activation of the MAPK/ERK pathway in macrophages, thus being critical for production of the proinflammatory cytokine TNF-alpha (TNF) during immune responses. Involved in the regulation of T-helper cell differentiation and IFNG expression in T-cells. Involved in mediating host resistance to bacterial infection through negative regulation of type I interferon (IFN) production. Transduces CD40 and TNFRSF1A signals that activate ERK in B-cells and macrophages, and thus may play a [...]*	*Nuclear factor NF-kappa-B p105 subunit; NF-kappa-B is a pleiotropic transcription factor present in almost all cell types and is the endpoint of a series of signal transduction events that are initiated by a vast array of stimuli related to many biological processes such as inflammation, immunity, differentiation, cell growth, tumorigenesis and apoptosis. NF-kappa-B is a homo- or heterodimeric complex formed by the Rel-like domain- containing proteins RELA/p65, RELB, NFKB1/p105, NFKB1/p50, REL and NFKB2/p52 and the heterodimeric p65-p50 complex appears to be most abundant one. The dime [...].*	0.993
**Map3k8**	Il6	ENSMUSP00000025078	ENSMUSP00000026845	*Mitogen-activated protein kinase kinase kinase 8; Required for lipopolysaccharide (LPS)-induced, TLR4-mediated activation of the MAPK/ERK pathway in macrophages, thus being critical for production of the proinflammatory cytokine TNF-alpha (TNF) during immune responses. Involved in the regulation of T-helper cell differentiation and IFNG expression in T-cells. Involved in mediating host resistance to bacterial infection through negative regulation of type I interferon (IFN) production. Transduces CD40 and TNFRSF1A signals that activate ERK in B-cells and macrophages, and thus may play a [...]*	*Interleukin-6; Cytokine with a wide variety of biological functions. It is a potent inducer of the acute phase response. Plays an essential role in the final differentiation of B-cells into Ig-secreting cells Involved in lymphocyte and monocyte differentiation. Acts on B-cells, T-cells, hepatocytes, hematopoietic progenitor cells and cells of the CNS. Required for the generation of T(H)17 cells. Also acts as a myokine. It is discharged into the bloodstream after muscle contraction and acts to increase the breakdown of fats and to improve insulin resistance*	0.689
**Map3k8**	BCL3	ENSMUSP00000025078	ENSMUSP00000113851	*Mitogen-activated protein kinase kinase kinase 8; Required for lipopolysaccharide (LPS)-induced, TLR4-mediated activation of the MAPK/ERK pathway in macrophages, thus being critical for*	*B-cell lymphoma 3 protein homolog; Contributes to the regulation of transcriptional activation of NF-kappa-B target genes. In the cytoplasm, inhibits the nuclear translocation of the NF-kappa-B p50 subunit (By similarity). In the nucleus,*	0.443

				production of the proinflammatory cytokine TNF-alpha (TNF) during immune responses. Involved in the regulation of T-helper cell differentiation and IFNG expression in T-cells. Involved in mediating host resistance to bacterial infection through negative regulation of type I interferon (IFN) production. Transduces CD40 and TNFRSF1A signals that activate ERK in B-cells and macrophages, and thus may play a [...]	*acts as transcriptional activator that promotes transcription of NF-kappa-B target genes. Contributes to the regulation of cell proliferation.*	
**Il6**	Tgfb1	ENSMUSP00000026845	ENSMUSP00000002678	*Interleukin-6; Cytokine with a wide variety of biological functions. It is a potent inducer of the acute phase response. Plays an essential role in the final differentiation of B-cells into Ig-secreting cells Involved in lymphocyte and monocyte differentiation. Acts on B-cells, T-cells, hepatocytes, hematopoietic progenitor cells and cells of the CNS. Required for the generation of T(H)17 cells. Also acts as a myokine. It is discharged into the bloodstream after muscle contraction and acts to increase the breakdown of fats and to improve insulin resistance. It induces myeloma and plasm [...]*	*Transforming growth factor beta-1 proprotein; Transforming growth factor beta-1 proprotein: Precursor of the Latency-associated peptide (LAP) and Transforming growth factor beta-1 (TGF-beta-1) chains, which constitute the regulatory and active subunit of TGF-beta-1, respectively. Transforming growth factor beta-1: Multifunctional protein that regulates the growth and differentiation of various cell types and is involved in various processes, such as normal development, immune function, microglia function and responses to neurodegeneration. Activation into mature form follows different [...]*	0.892
**Il6**	Nlrp12	ENSMUSP00000026845	ENSMUSP00000104293	*Interleukin-6; Cytokine with a wide variety of biological functions. It is a potent inducer of the acute phase response. Plays an essential role in the final differentiation of B-cells into Ig-secreting cells Involved in lymphocyte and monocyte differentiation. Acts on B-cells, T-cells, hepatocytes, hematopoietic progenitor cells and cells of the CNS. Required for the generation of T(H)17 cells. Also acts as a myokine. It is discharged into the bloodstream after muscle contraction and acts to increase the breakdown of fats and to improve insulin resistance. It induces myeloma and plasm [...]*	*NACHT, LRR and PYD domains-containing protein 12; Plays an essential role as an potent mitigator of inflammation. Primarily expressed in dendritic cells and macrophages, inhibits both canonical and non- canonical NF-kappa-B and ERK activation pathways. Functions as a negative regulator of NOD2 by targeting it to degradation via the proteasome pathway. In turn, promotes bacterial tolerance. Inhibits also the DDX58-mediated immune signaling against RNA viruses by reducing the E3 ubiquitin ligase TRIM25-mediated 'Lys-63'-linked DDX58 activation but enhancing the E3 ubiquitin ligase RNF125 [...]*	0.407
**Il6**	Nkfb1	ENSMUSP00000026845	ENSMUSP00000029812	*Interleukin-6; Cytokine with a wide variety of biological functions. It is a potent inducer of the acute phase response. Plays an essential role in the final differentiation of B-cells into Ig-secreting cells Involved in lymphocyte and monocyte differentiation. Acts on B-cells, T-cells, hepatocytes, hematopoietic progenitor cells and cells of the CNS. Required for the generation of T(H)17 cells. Also acts as a myokine. It is discharged into the bloodstream after muscle contraction and acts to increase the breakdown of fats and to improve insulin resistance. It induces myeloma and plasm [...]*	*Nuclear factor NF-kappa-B p105 subunit; NF-kappa-B is a pleiotropic transcription factor present in almost all cell types and is the endpoint of a series of signal transduction events that are initiated by a vast array of stimuli related to many biological processes such as inflammation, immunity, differentiation, cell growth, tumorigenesis and apoptosis. NF-kappa-B is a homo- or heterodimeric complex formed by the Rel-like domain- containing proteins RELA/p65, RELB, NFKB1/p105, NFKB1/p50, REL and NFKB2/p52 and the heterodimeric p65-p50 complex appears to be most abundant one. The dime [...].*	0.986
**Il6**	Map3k8	ENSMUSP00000026845	ENSMUSP00000025078	*Interleukin-6; Cytokine with a wide variety of biological functions. It is a potent inducer of the*	*Mitogen-activated protein kinase kinase kinase 8; Required for lipopolysaccharide (LPS)-induced, TLR4-mediated*	0.689

				acute phase response. Plays an essential role in the final differentiation of B-cells into Ig-secreting cells Involved in lymphocyte and monocyte differentiation. Acts on B-cells, T-cells, hepatocytes, hematopoietic progenitor cells and cells of the CNS. Required for the generation of T(H)17 cells. Also acts as a myokine. It is discharged into the bloodstream after muscle contraction and acts to increase the breakdown of fats and to improve insulin resistance. It induces myeloma and plasm [...]	*activation of the MAPK/ERK pathway in macrophages, thus being critical for production of the proinflammatory cytokine TNF-alpha (TNF) during immune responses. Involved in the regulation of T-helper cell differentiation and IFNG expression in T-cells. Involved in mediating host resistance to bacterial infection through negative regulation of type I interferon (IFN) production. Transduces CD40 and TNFRSF1A signals that activate ERK in B-cells and macrophages, and thus may play a [...]*	
**Il6**	Cxcl3	ENSMUSP00000026845	ENSMUSP00000031326	*Interleukin-6; Cytokine with a wide variety of biological functions. It is a potent inducer of the acute phase response. Plays an essential role in the final differentiation of B-cells into Ig-secreting cells Involved in lymphocyte and monocyte differentiation. Acts on B-cells, T-cells, hepatocytes, hematopoietic progenitor cells and cells of the CNS. Required for the generation of T(H)17 cells. Also acts as a myokine. It is discharged into the bloodstream after muscle contraction and acts to increase the breakdown of fats and to improve insulin resistance. It induces myeloma and plasm [...]*	*C-X-C motif chemokine 3; Ligand for CXCR2. Has chemotactic activity for neutrophils. May play a role in inflammation and exert its effects on endothelial cells in an autocrine fashion.*	0.706
**Il6**	Cxcl2	ENSMUSP00000026845	ENSMUSP00000074885	*Interleukin-6; Cytokine with a wide variety of biological functions. It is a potent inducer of the acute phase response. Plays an essential role in the final differentiation of B-cells into Ig-secreting cells Involved in lymphocyte and monocyte differentiation. Acts on B-cells, T-cells, hepatocytes, hematopoietic progenitor cells and cells of the CNS. Required for the generation of T(H)17 cells. Also acts as a myokine. It is discharged into the bloodstream after muscle contraction and acts to increase the breakdown of fats and to improve insulin resistance. It induces myeloma and plasm [...]*	*C-X-C motif chemokine 2; Chemotactic for human polymorphonuclear leukocytes but does not induce chemokinesis or an oxidative burst.*	0.936
**Il6**	BCL3	ENSMUSP00000026845	ENSMUSP00000113851	*Interleukin-6; Cytokine with a wide variety of biological functions. It is a potent inducer of the acute phase response. Plays an essential role in the final differentiation of B-cells into Ig-secreting cells Involved in lymphocyte and monocyte differentiation. Acts on B-cells, T-cells, hepatocytes, hematopoietic progenitor cells and cells of the CNS. Required for the generation of T(H)17 cells. Also acts as a myokine. It is discharged into the bloodstream after muscle contraction and acts to increase the breakdown of fats and to improve insulin resistance. It induces myeloma and plasm [...]*	*B-cell lymphoma 3 protein homolog; Contributes to the regulation of transcriptional activation of NF-kappa-B target genes. In the cytoplasm, inhibits the nuclear translocation of the NF-kappa-B p50 subunit (By similarity). In the nucleus, acts as transcriptional activator that promotes transcription of NF-kappa-B target genes. Contributes to the regulation of cell proliferation*	0.697

**Cxcl3**	Nkfb1	ENSMUSP00000031326	ENSMUSP00000029812	*C-X-C motif chemokine 3; Ligand for CXCR2. Has chemotactic activity for neutrophils. May play a role in inflammation and exert its effects on endothelial cells in an autocrine fashion.*	*Nuclear factor NF-kappa-B p105 subunit; NF-kappa-B is a pleiotropic transcription factor present in almost all cell types and is the endpoint of a series of signal transduction events that are initiated by a vast array of stimuli related to many biological processes such as inflammation, immunity, differentiation, cell growth, tumorigenesis and apoptosis. NF-kappa-B is a homo- or heterodimeric complex formed by the Rel-like domain- containing proteins RELA/p65, RELB, NFKB1/p105, NFKB1/p50, REL and NFKB2/p52 and the heterodimeric p65-p50 complex appears to be most abundant one. The dime [...]*	0.917
**Cxcl3**	Il6	ENSMUSP00000031326	ENSMUSP00000026845	*C-X-C motif chemokine 3; Ligand for CXCR2. Has chemotactic activity for neutrophils. May play a role in inflammation and exert its effects on endothelial cells in an autocrine fashion.*	*Interleukin-6; Cytokine with a wide variety of biological functions. It is a potent inducer of the acute phase response. Plays an essential role in the final differentiation of B-cells into Ig-secreting cells Involved in lymphocyte and monocyte differentiation. Acts on B-cells, T-cells, hepatocytes, hematopoietic progenitor cells and cells of the CNS. Required for the generation of T(H)17 cells. Also acts as a myokine. It is discharged into the bloodstream after muscle contraction and acts to increase the breakdown of fats and to improve insulin resistance. It induces myeloma and plasm [...]*	0.706
**Cxcl3**	Cxcl2	ENSMUSP00000031326	ENSMUSP00000074885	*C-X-C motif chemokine 3; Ligand for CXCR2. Has chemotactic activity for neutrophils. May play a role in inflammation and exert its effects on endothelial cells in an autocrine fashion.*	*C-X-C motif chemokine 2; Chemotactic for human polymorphonuclear leukocytes but does not induce chemokinesis or an oxidative burst.*	0.957
**Cxcl2**	Tgfb1	ENSMUSP00000074885	ENSMUSP00000002678	*C-X-C motif chemokine 2; Chemotactic for human polymorphonuclear leukocytes but does not induce chemokinesis or an oxidative burst.*	*Transforming growth factor beta-1 proprotein; Transforming growth factor beta-1 proprotein: Precursor of the Latency-associated peptide (LAP) and Transforming growth factor beta-1 (TGF-beta-1) chains, which constitute the regulatory and active subunit of TGF-beta-1, respectively. Transforming growth factor beta-1: Multifunctional protein that regulates the growth and differentiation of various cell types and is involved in various processes, such as normal development, immune function, microglia function and responses to neurodegeneration. Activation into mature form follows different [...]*	0.499
**Cxcl2**	Nfkb1	ENSMUSP00000074885	ENSMUSP00000029812	*C-X-C motif chemokine 2; Chemotactic for human polymorphonuclear leukocytes but does not induce chemokinesis or an oxidative burst.*	*Nuclear factor NF-kappa-B p105 subunit; NF-kappa-B is a pleiotropic transcription factor present in almost all cell types and is the endpoint of a series of signal transduction events that are initiated by a vast array of stimuli related to many biological processes such as inflammation, immunity, differentiation, cell growth, tumorigenesis and apoptosis. NF-kappa-B is a homo- or heterodimeric complex formed by the Rel-like domain- containing proteins RELA/p65, RELB, NFKB1/p105, NFKB1/p50, REL and NFKB2/p52 and the heterodimeric p65-p50 complex appears to be most abundant one. The dime [...]*	0.938

**Cxcl2**	IL6	ENSMUSP00000074885	ENSMUSP00000026845	*C-X-C motif chemokine 2; Chemotactic for human polymorphonuclear leukocytes but does not induce chemokinesis or an oxidative burst.*	*Interleukin-6; Cytokine with a wide variety of biological functions. It is a potent inducer of the acute phase response. Plays an essential role in the final differentiation of B-cells into Ig-secreting cells Involved in lymphocyte and monocyte differentiation. Acts on B-cells, T-cells, hepatocytes, hematopoietic progenitor cells and cells of the CNS. Required for the generation of T(H)17 cells. Also acts as a myokine. It is discharged into the bloodstream after muscle contraction and acts to increase the breakdown of fats and to improve insulin resistance. It induces myeloma and plasm [...]*	0.936
**Cxcl2**	Cxcl3	ENSMUSP00000074885	ENSMUSP00000031326	*C-X-C motif chemokine 2; Chemotactic for human polymorphonuclear leukocytes but does not induce chemokinesis or an oxidative burst.*	*C-X-C motif chemokine 3; Ligand for CXCR2. Has chemotactic activity for neutrophils. May play a role in inflammation and exert its effects on endothelial cells in an autocrine fashion.*	0.957
**BCL3**	Tgfb1	ENSMUSP00000113851	ENSMUSP00000002678	*B-cell lymphoma 3 protein homolog; Contributes to the regulation of transcriptional activation of NF-kappa-B target genes. In the cytoplasm, inhibits the nuclear translocation of the NF-kappa-B p50 subunit (By similarity). In the nucleus, acts as transcriptional activator that promotes transcription of NF-kappa-B target genes. Contributes to the regulation of cell proliferation.*	*Transforming growth factor beta-1 proprotein; Transforming growth factor beta-1 proprotein: Precursor of the Latency-associated peptide (LAP) and Transforming growth factor beta-1 (TGF-beta-1) chains, which constitute the regulatory and active subunit of TGF-beta-1, respectively. Transforming growth factor beta-1: Multifunctional protein that regulates the growth and differentiation of various cell types and is involved in various processes, such as normal development, immune function, microglia function and responses to neurodegeneration. Activation into mature form follows different [...]*	0.553
**BCL3**	Nfkb1	ENSMUSP00000113851	ENSMUSP00000029812	*B-cell lymphoma 3 protein homolog; Contributes to the regulation of transcriptional activation of NF-kappa-B target genes. In the cytoplasm, inhibits the nuclear translocation of the NF-kappa-B p50 subunit (By similarity). In the nucleus, acts as transcriptional activator that promotes transcription of NF-kappa-B target genes. Contributes to the regulation of cell proliferation.*	*B-cell lymphoma 3 protein homolog; Contributes to the regulation of transcriptional activation of NF-kappa-B target genes. In the cytoplasm, inhibits the nuclear translocation of the NF-kappa-B p50 subunit (By similarity). In the nucleus, acts as transcriptional activator that promotes transcription of NF-kappa-B target genes. Contributes to the regulation of cell proliferation*	0.999
**BCL3**	Map3k8	ENSMUSP00000113851	ENSMUSP00000025078	*B-cell lymphoma 3 protein homolog; Contributes to the regulation of transcriptional activation of NF-kappa-B target genes. In the cytoplasm, inhibits the nuclear translocation of the NF-kappa-B p50 subunit (By similarity). In the nucleus, acts as transcriptional activator that promotes transcription of NF-kappa-B target genes. Contributes to the regulation of cell proliferation.*	*Mitogen-activated protein kinase kinase kinase 8; Required for lipopolysaccharide (LPS)-induced, TLR4-mediated activation of the MAPK/ERK pathway in macrophages, thus being critical for production of the proinflammatory cytokine TNF-alpha (TNF) during immune responses. Involved in the regulation of T-helper cell differentiation and IFNG expression in T-cells. Involved in mediating host resistance to bacterial infection through negative regulation of type I interferon (IFN) production. Transduces CD40 and TNFRSF1A signals that activate ERK in B-cells and macrophages, and thus may play a [...]*	0.443
**BCL3**	IL6	ENSMUSP00000113851	ENSMUSP00000026845	*B-cell lymphoma 3 protein homolog; Contributes to the regulation of transcriptional activation of NF-kappa-B target genes. In the cytoplasm, inhibits the nuclear translocation of the NF-kappa-B p50 subunit (By similarity). In the nucleus, acts as*	*Interleukin-6; Cytokine with a wide variety of biological functions. It is a potent inducer of the acute phase response. Plays an essential role in the final differentiation of B-cells into Ig-secreting cells Involved in lymphocyte and monocyte differentiation. Acts on B-cells, T-cells, hepatocytes,*	0.697

		transcriptional activator that promotes transcription of NF-kappa-B target genes. Contributes to the regulation of cell proliferation.	*hematopoietic progenitor cells and cells of the CNS. Required for the generation of T(H)17 cells. Also acts as a myokine. It is discharged into the bloodstream after muscle contraction and acts to increase the breakdown of fats and to improve insulin resistance. It induces myeloma and plasm [...]*

Table S3: Network Interaction Scores of Apoptotic Pathway Genes in Protein-Protein Interactions

node1	node2	node1 accession	node2 accession	node1 annotation	node2 annotation	score
**BAX**	BCL2	ENSP00000293288	ENSP00000381185	Apoptosis regulator BAX; Plays a role in the mitochondrial apoptotic process. Under normal conditions, BAX is largely cytosolic via constant retrotranslocation from mitochondria to th cytosol mediated by BCL2L1/Bcl-xL, which avoids accumulatic of toxic BAX levels at the mitochondrial outer membrane (MOM). Under stress conditions, undergoes a conformation change that causes translocation to the mitochondrion membrane leading to the release of cytochrome c that then triggers apoptosi Promotes activation of CASP3, and thereby apoptosis.	Apoptosis regulator Bcl-2; Suppresses apoptosis in a variety of cell systems including factor-dependent lymphohematopoietic and neural cells. Regulates cell death by controlling the mitochondrial membrane permeability. Appears to function in a feedback loop system with caspases. Inhibits caspase activity either by preventing the release of cytochrome c from the mitochondria and/or by binding to the apoptosis-activating factor (APAF-1). May attenuate inflammation by impairing NLRP1-inflammasome activation, hence CASP1 activation and IL1B release.	0.999
**BAX**	CASP3	ENSP00000293288	ENSP00000311032	Apoptosis regulator BAX; Plays a role in the mitochondrial apoptotic process. Under normal conditions, BAX is largely cytosolic via constant retrotranslocation from mitochondria to the cytosol mediated by BCL2L1/Bcl-xL, which avoids accumulation of toxic BAX levels at the mitochondrial outer membrane (MOM). Under stress conditions, undergoes a conformation change that causes translocation to the mitochondrion membrane, leading to the release of cytochrome c that then triggers apoptosis. Promotes activation of CASP3, and thereby apoptosis.	Caspase-3 subunit p12; Involved in the activation cascade of caspases responsible for apoptosis execution. At the onset of apoptosis it proteolytically cleaves poly(ADP-ribose) polymerase (PARP) at a '216-Asp-|-Gly-217' bond. Cleaves and activates sterol regulatory element binding proteins (SREBPs) between the basic helix-loop-helix leucine zipper domain and the membrane attachment domain. Cleaves and activates caspase-6, -7 and -9. Involved in the cleavage of huntingtin. Triggers cell adhesion in sympathetic neurons through RET cleavage.	0.884
**BAX**	CYCS	ENSP00000293288	ENSP00000307786	Apoptosis regulator BAX; Plays a role in the mitochondrial apoptotic process. Under normal conditions, BAX is largely cytosolic via constant retrotranslocation from mitochondria to the cytosol mediated by BCL2L1/Bcl-xL, which avoids accumulation of toxic BAX levels at the mitochondrial outer membrane (MOM). Under stress conditions, undergoes a conformation change that causes translocation to the mitochondrion membrane, leading to the release of cytochrome c that then triggers apoptosis. Promotes activation of CASP3, and thereby apoptosis.	Cytochrome c; Electron carrier protein. The oxidized form of the cytochrome c heme group can accept an electron from the heme group of the cytochrome c1 subunit of cytochrome reductase. Cytochrome c then transfers this electron to the cytochrome oxidase complex, the final protein carrier in the mitochondrial electron-transport chain.	0.993
**BAX**	TP53	ENSP00000293288	ENSP00000269305	Apoptosis regulator BAX; Plays a role in the mitochondrial apoptotic process. Under normal conditions, BAX is largely cytosolic via constant retrotranslocation from mitochondria to the cytosol mediated by BCL2L1/Bcl-xL, which avoids accumulation of toxic BAX levels at the mitochondrial outer membrane (MOM). Under stress conditions, undergoes a conformation change that causes translocation to the mitochondrion membrane, leading to the release of cytochrome c that then triggers apoptosis. Promotes activation of CASP3, and thereby apoptosis.	Cellular tumor antigen p53; Acts as a tumor suppressor in many tumor types; induces growth arrest or apoptosis depending on the physiological circumstances and cell type. Involved in cell cycle regulation as a trans-activator that acts to negatively regulate cell division by controlling a set of genes required for this process. One of the activated genes is an inhibitor of cyclin-dependent kinases. Apoptosis induction seems to be mediated either by stimulation of BAX and FAS antigen expression, or by repression of Bcl-2 expression. Its pro-apoptotic activity is activated via its intera [...]	0.994
**BCL2**	BAX	ENSP00000381185	ENSP00000293288	Apoptosis regulator Bcl-2; Suppresses apoptosis in a variety of cell systems including factor-dependent lymphohematopoietic and neural cells. Regulates cell death by controlling the mitochondrial membrane permeability. Appears to function in a feedback loop system with caspases. Inhibits caspase activity either by preventing the release of cytochrome c from the mitochondria and/or by binding to the apoptosis-activating factor (APAF-1). May attenuate inflammation by impairing	Apoptosis regulator BAX; Plays a role in the mitochondrial apoptotic process. Under normal conditions, BAX is largely cytosolic via constant retrotranslocation from mitochondria to the cytosol mediated by BCL2L1/Bcl-xL, which avoids accumulation of toxic BAX levels at the mitochondrial outer membrane (MOM). Under stress conditions, undergoes a conformation change that causes translocation to the mitochondrion membrane, leading to the	0.999

				NLRP1-inflammasome activation, hence CASP1 activation and IL1B release.	release of cytochrome c that then triggers apoptosis. Promotes activation of CASP3, and thereby apoptosis.	
**BCL2**	CASP3	ENSP00000381185	ENSP00000311032	Apoptosis regulator Bcl-2; Suppresses apoptosis in a variety of cell systems including factor-dependent lymphohematopoietic and neural cells. Regulates cell death by controlling the mitochondrial membrane permeability. Appears to function in a feedback loop system with caspases. Inhibits caspase activity either by preventing the release of cytochrome c from the mitochondria and/or by binding to the apoptosis-activating factor (APAF-1). May attenuate inflammation by impairing NLRP1-inflammasome activation, hence CASP1 activation and IL1B release.	Caspase-3 subunit p12; Involved in the activation cascade of caspases responsible for apoptosis execution. At the onset of apoptosis it proteolytically cleaves poly(ADP-ribose) polymerase (PARP) at a '216-Asp-|-Gly-217' bond. Cleaves and activates sterol regulatory element binding proteins (SREBPs) between the basic helix-loop-helix leucine zipper domain and the membrane attachment domain. Cleaves and activates caspase-6, -7 and -9. Involved in the cleavage of huntingtin. Triggers cell adhesion in sympathetic neurons through RET cleavage.	0.987
**BCL2**	CYCS	ENSP00000381185	ENSP00000307786	Apoptosis regulator Bcl-2; Suppresses apoptosis in a variety of cell systems including factor-dependent lymphohematopoietic and neural cells. Regulates cell death by controlling the mitochondrial membrane permeability. Appears to function in a feedback loop system with caspases. Inhibits caspase activity either by preventing the release of cytochrome c from the mitochondria and/or by binding to the apoptosis-activating factor (APAF-1). May attenuate inflammation by impairing NLRP1-inflammasome activation, hence CASP1 activation and IL1B release.	Cytochrome c; Electron carrier protein. The oxidized form of the cytochrome c heme group can accept an electron from the heme group of the cytochrome c1 subunit of cytochrome reductase. Cytochrome c then transfers this electron to the cytochrome oxidase complex, the final protein carrier in the mitochondrial electron-transport chain.	0.997
**BCL2**	TP53	ENSP00000381185	ENSP00000269305	Apoptosis regulator Bcl-2; Suppresses apoptosis in a variety of cell systems including factor-dependent lymphohematopoietic and neural cells. Regulates cell death by controlling the mitochondrial membrane permeability. Appears to function in a feedback loop system with caspases. Inhibits caspase activity either by preventing the release of cytochrome c from the mitochondria and/or by binding to the apoptosis-activating factor (APAF-1). May attenuate inflammation by impairing NLRP1-inflammasome activation, hence CASP1 activation and IL1B release.	Cellular tumor antigen p53; Acts as a tumor suppressor in many tumor types; induces growth arrest or apoptosis depending on the physiological circumstances and cell type. Involved in cell cycle regulation as a trans-activator that acts to negatively regulate cell division by controlling a set of genes required for this process. One of the activated genes is an inhibitor of cyclin-dependent kinases. Apoptosis induction seems to be mediated either by stimulation of BAX and FAS antigen expression, or by repression of Bcl-2 expression. Its pro-apoptotic activity is activated via its intera [...]	0.999
**CASP3**	BAX	ENSP00000311032	ENSP00000293288	Caspase-3 subunit p12; Involved in the activation cascade of caspases responsible for apoptosis execution. At the onset of apoptosis it proteolytically cleaves poly(ADP-ribose) polymerase (PARP) at a '216-Asp-|-Gly-217' bond. Cleaves and activates sterol regulatory element binding proteins (SREBPs) between the basic helix-loop-helix leucine zipper domain and the membrane attachment domain. Cleaves and activates caspase-6, -7 and -9. Involved in the cleavage of huntingtin. Triggers cell adhesion in sympathetic neurons through RET cleavage.	Apoptosis regulator BAX; Plays a role in the mitochondrial apoptotic process. Under normal conditions, BAX is largely cytosolic via constant retrotranslocation from mitochondria to the cytosol mediated by BCL2L1/Bcl-xL, which avoids accumulation of toxic BAX levels at the mitochondrial outer membrane (MOM). Under stress conditions, undergoes a conformation change that causes translocation to the mitochondrion membrane, leading to the release of cytochrome c that then triggers apoptosis. Promotes activation of CASP3, and thereby apoptosis.	0.884
**CASP3**	BCL2	ENSP00000311032	ENSP00000381185	Caspase-3 subunit p12; Involved in the activation cascade of caspases responsible for apoptosis execution. At the onset of apoptosis it proteolytically cleaves poly(ADP-ribose) polymerase (PARP) at a '216-Asp-|-Gly-217' bond. Cleaves and activates sterol regulatory element binding proteins (SREBPs) between the basic helix-loop-helix leucine zipper domain and the membrane attachment domain. Cleaves and activates caspase-6, -7 and -9. Involved in the cleavage of	Apoptosis regulator Bcl-2; Suppresses apoptosis in a variety of cell systems including factor-dependent lymphohematopoietic and neural cells. Regulates cell death by controlling the mitochondrial membrane permeability. Appears to function in a feedback loop system with caspases. Inhibits caspase activity either by preventing the release of cytochrome c from the mitochondria and/or by binding to the apoptosis-activating factor (APAF-1). May attenuate	0.987

				huntingtin. Triggers cell adhesion in sympathetic neurons through RET cleavage.	inflammation by impairing NLRP1-inflammasome activation, hence CASP1 activation and IL1B release.	
**CASP3**	CYCS	ENSP00000311032	ENSP00000307786	Caspase-3 subunit p12; Involved in the activation cascade of caspases responsible for apoptosis execution. At the onset of apoptosis it proteolytically cleaves poly(ADP-ribose) polymerase (PARP) at a '216-Asp-|-Gly-217' bond. Cleaves and activates sterol regulatory element binding proteins (SREBPs) between the basic helix-loop-helix leucine zipper domain and the membrane attachment domain. Cleaves and activates caspase-6, -7 and -9. Involved in the cleavage of huntingtin. Triggers cell adhesion in sympathetic neurons through RET cleavage.	Cytochrome c; Electron carrier protein. The oxidized form of the cytochrome c heme group can accept an electron from the heme group of the cytochrome c1 subunit of cytochrome reductase. Cytochrome c then transfers this electron to the cytochrome oxidase complex, the final protein carrier in the mitochondrial electron-transport chain.	0.997
**CASP3**	TP53	ENSP00000311032	ENSP00000269305	Caspase-3 subunit p12; Involved in the activation cascade of caspases responsible for apoptosis execution. At the onset of apoptosis it proteolytically cleaves poly(ADP-ribose) polymerase (PARP) at a '216-Asp-|-Gly-217' bond. Cleaves and activates sterol regulatory element binding proteins (SREBPs) between the basic helix-loop-helix leucine zipper domain and the membrane attachment domain. Cleaves and activates caspase-6, -7 and -9. Involved in the cleavage of huntingtin. Triggers cell adhesion in sympathetic neurons through RET cleavage.	Cellular tumor antigen p53; Acts as a tumor suppressor in many tumor types; induces growth arrest or apoptosis depending on the physiological circumstances and cell type. Involved in cell cycle regulation as a trans-activator that acts to negatively regulate cell division by controlling a set of genes required for this process. One of the activated genes is an inhibitor of cyclin-dependent kinases. Apoptosis induction seems to be mediated either by stimulation of BAX and FAS antigen expression, or by repression of Bcl-2 expression. Its pro-apoptotic activity is activated via its intera [...]	0.956
**CYCS**	BAX	ENSP00000307786	ENSP00000293288	Cytochrome c; Electron carrier protein. The oxidized form of the cytochrome c heme group can accept an electron from the heme group of the cytochrome c1 subunit of cytochrome reductase. Cytochrome c then transfers this electron to the cytochrome oxidase complex, the final protein carrier in the mitochondrial electron-transport chain.	Apoptosis regulator BAX; Plays a role in the mitochondrial apoptotic process. Under normal conditions, BAX is largely cytosolic via constant retrotranslocation from mitochondria to the cytosol mediated by BCL2L1/Bcl-xL, which avoids accumulation of toxic BAX levels at the mitochondrial outer membrane (MOM). Under stress conditions, undergoes a conformation change that causes translocation to the mitochondrion membrane, leading to the release of cytochrome c that then triggers apoptosis. Promotes activation of CASP3, and thereby apoptosis.	0.993
**CYCS**	BCL2	ENSP00000307786	ENSP00000381185	Cytochrome c; Electron carrier protein. The oxidized form of the cytochrome c heme group can accept an electron from the heme group of the cytochrome c1 subunit of cytochrome reductase. Cytochrome c then transfers this electron to the cytochrome oxidase complex, the final protein carrier in the mitochondrial electron-transport chain.	Apoptosis regulator Bcl-2; Suppresses apoptosis in a variety of cell systems including factor-dependent lymphohematopoietic and neural cells. Regulates cell death by controlling the mitochondrial membrane permeability. Appears to function in a feedback loop system with caspases. Inhibits caspase activity either by preventing the release of cytochrome c from the mitochondria and/or by binding to the apoptosis-activating factor (APAF-1). May attenuate inflammation by impairing NLRP1-inflammasome activation, hence CASP1 activation and IL1B release.	0.997
**CYCS**	CASP3	ENSP00000307786	ENSP00000311032	Cytochrome c; Electron carrier protein. The oxidized form of the cytochrome c heme group can accept an electron from the heme group of the cytochrome c1 subunit of cytochrome reductase. Cytochrome c then transfers this electron to the cytochrome oxidase complex, the final protein carrier in the mitochondrial electron-transport chain.	Caspase-3 subunit p12; Involved in the activation cascade of caspases responsible for apoptosis execution. At the onset of apoptosis it proteolytically cleaves poly(ADP-ribose) polymerase (PARP) at a '216-Asp-|-Gly-217' bond. Cleaves and activates sterol regulatory element binding proteins (SREBPs) between the basic helix-loop-helix leucine zipper domain and the membrane attachment domain. Cleaves and activates caspase-6, -7 and -9. Involved in the cleavage of huntingtin. Triggers cell adhesion in sympathetic neurons through RET cleavage.	0.997

**CYCS**	TP53	ENSP00000307786	ENSP00000269305	Cytochrome c; Electron carrier protein. The oxidized form of the cytochrome c heme group can accept an electron from the heme group of the cytochrome c1 subunit of cytochrome reductase. Cytochrome c then transfers this electron to the cytochrome oxidase complex, the final protein carrier in the mitochondrial electron-transport chain.	Cellular tumor antigen p53; Acts as a tumor suppressor in many tumor types; induces growth arrest or apoptosis depending on the physiological circumstances and cell type. Involved in cell cycle regulation as a trans-activator that acts to negatively regulate cell division by controlling a set of genes required for this process. One of the activated genes is an inhibitor of cyclin-dependent kinases. Apoptosis induction seems to be mediated either by stimulation of BAX and FAS antigen expression, or by repression of Bcl-2 expression. Its pro-apoptotic activity is activated via its intera [...]	0.911
**TP53**	BAX	ENSP00000269305	ENSP00000293288	Cellular tumor antigen p53; Acts as a tumor suppressor in many tumor types; induces growth arrest or apoptosis depending on the physiological circumstances and cell type. Involved in cell cycle regulation as a trans-activator that acts to negatively regulate cell division by controlling a set of genes required for this process. One of the activated genes is an inhibitor of cyclin-dependent kinases. Apoptosis induction seems to be mediated either by stimulation of BAX and FAS antigen expression, or by repression of Bcl-2 expression. Its pro-apoptotic activity is activated via its intera [...]	Apoptosis regulator BAX; Plays a role in the mitochondrial apoptotic process. Under normal conditions, BAX is largely cytosolic via constant retrotranslocation from mitochondria to the cytosol mediated by BCL2L1/Bcl-xL, which avoids accumulation of toxic BAX levels at the mitochondrial outer membrane (MOM). Under stress conditions, undergoes a conformation change that causes translocation to the mitochondrion membrane, leading to the release of cytochrome c that then triggers apoptosis. Promotes activation of CASP3, and thereby apoptosis.	0.994
**TP53**	BCL2	ENSP00000269305	ENSP00000381185	Cellular tumor antigen p53; Acts as a tumor suppressor in many tumor types; induces growth arrest or apoptosis depending on the physiological circumstances and cell type. Involved in cell cycle regulation as a trans-activator that acts to negatively regulate cell division by controlling a set of genes required for this process. One of the activated genes is an inhibitor of cyclin-dependent kinases. Apoptosis induction seems to be mediated either by stimulation of BAX and FAS antigen expression, or by repression of Bcl-2 expression. Its pro-apoptotic activity is activated via its intera [...]	Apoptosis regulator Bcl-2; Suppresses apoptosis in a variety of cell systems including factor-dependent lymphohematopoietic and neural cells. Regulates cell death by controlling the mitochondrial membrane permeability. Appears to function in a feedback loop system with caspases. Inhibits caspase activity either by preventing the release of cytochrome c from the mitochondria and/or by binding to the apoptosis-activating factor (APAF-1). May attenuate inflammation by impairing NLRP1-inflammasome activation, hence CASP1 activation and IL1B release.	0.999
**TP53**	CASP3	ENSP00000269305	ENSP00000311032	Cellular tumor antigen p53; Acts as a tumor suppressor in many tumor types; induces growth arrest or apoptosis depending on the physiological circumstances and cell type. Involved in cell cycle regulation as a trans-activator that acts to negatively regulate cell division by controlling a set of genes required for this process. One of the activated genes is an inhibitor of cyclin-dependent kinases. Apoptosis induction seems to be mediated either by stimulation of BAX and FAS antigen expression, or by repression of Bcl-2 expression. Its pro-apoptotic activity is activated via its intera [...]	Caspase-3 subunit p12; Involved in the activation cascade of caspases responsible for apoptosis execution. At the onset of apoptosis it proteolytically cleaves poly(ADP-ribose) polymerase (PARP) at a '216-Asp-|-Gly-217' bond. Cleaves and activates sterol regulatory element binding proteins (SREBPs) between the basic helix-loop-helix leucine zipper domain and the membrane attachment domain. Cleaves and activates caspase-6, -7 and -9. Involved in the cleavage of huntingtin. Triggers cell adhesion in sympathetic neurons through RET cleavage.	0.956
**TP53**	CYCS	ENSP00000269305	ENSP00000307786	Cellular tumor antigen p53; Acts as a tumor suppressor in many tumor types; induces growth arrest or apoptosis depending on the physiological circumstances and cell type. Involved in cell cycle regulation as a trans-activator that acts to negatively regulate cell division by controlling a set of genes required for this process. One of the activated genes is an inhibitor of cyclin-dependent kinases. Apoptosis induction seems to be mediated either by stimulation of BAX and FAS antigen expression, or by repression of Bcl-2 expression. Its pro-apoptotic activity is activated via its intera [...]	Cytochrome c; Electron carrier protein. The oxidized form of the cytochrome c heme group can accept an electron from the heme group of the cytochrome c1 subunit of cytochrome reductase. Cytochrome c then transfers this electron to the cytochrome oxidase complex, the final protein carrier in the mitochondrial electron-transport chain.	0.911
